# Dynamic Functional Connectivity Alterations and Their Associated Gene Expression Pattern in Autism Spectrum Disorders

**DOI:** 10.3389/fnins.2021.794151

**Published:** 2022-01-10

**Authors:** Lin Ma, Tengfei Yuan, Wei Li, Lining Guo, Dan Zhu, Zirui Wang, Zhixuan Liu, Kaizhong Xue, Yaoyi Wang, Jiawei Liu, Weiqi Man, Zhaoxiang Ye, Feng Liu, Junping Wang

**Affiliations:** ^1^Department of Radiology and Tianjin Key Laboratory of Functional Imaging, Tianjin Medical University General Hospital, Tianjin, China; ^2^Department of Radiology, Tianjin Medical University Cancer Institute and Hospital, National Clinical Research Center for Cancer, Key Laboratory of Cancer Prevention and Therapy, Tianjin’s Clinical Research Center for Cancer, Tianjin, China; ^3^Department of Radiology, Tianjin Medical University General Hospital Airport Hospital, Tianjin, China

**Keywords:** Allen Human Brain Atlas, transcriptome, autism spectrum disorders, neuroimaging, dynamic functional connectivity

## Abstract

Autism spectrum disorders (ASDs) are a group of heterogeneous neurodevelopmental disorders that are highly heritable and are associated with impaired dynamic functional connectivity (DFC). However, the molecular mechanisms behind DFC alterations remain largely unknown. Eighty-eight patients with ASDs and 87 demographically matched typical controls (TCs) from the Autism Brain Imaging Data Exchange II database were included in this study. A seed-based sliding window approach was then performed to investigate the DFC changes in each of the 29 seeds in 10 classic resting-state functional networks and the whole brain. Subsequently, the relationships between DFC alterations in patients with ASDs and their symptom severity were assessed. Finally, transcription-neuroimaging association analyses were conducted to explore the molecular mechanisms of DFC disruptions in patients with ASDs. Compared with TCs, patients with ASDs showed significantly increased DFC between the right dorsolateral prefrontal cortex (DLPFC) and left fusiform/lingual gyrus, between the DLPFC and the superior temporal gyrus, between the right frontal eye field (FEF) and left middle frontal gyrus, between the FEF and the right angular gyrus, and between the left intraparietal sulcus and the right middle temporal gyrus. Moreover, significant relationships between DFC alterations and symptom severity were observed. Furthermore, the genes associated with DFC changes in ASDs were identified by performing gene-wise across-sample spatial correlation analysis between gene expression extracted from six donors’ brain of the Allen Human Brain Atlas and case-control DFC difference. In enrichment analysis, these genes were enriched for processes associated with synaptic signaling and voltage-gated ion channels and calcium pathways; also, these genes were highly expressed in autistic disorder, chronic alcoholic intoxication and several disorders related to depression. These results not only demonstrated higher DFC in patients with ASDs but also provided novel insight into the molecular mechanisms underlying these alterations.

## Introduction

Autism spectrum disorders (ASDs) are a group of heterogeneous neurodevelopmental disorders characterized by social communication defects, stereotyped behaviors and restricted interests or activities (Lai et al., 2014). The increasing global prevalence of ASDs in recent years is consistent across different data sources (Morales-Hidalgo et al., 2018). Neuroimaging techniques have been used to characterize the complex biomarkers of these disorders and a great deal of evidence supports the aberrant functional connectivity (FC) of various cortical networks. However, most previous studies used traditional “static” approaches based on resting-state functional magnetic resonance imaging (rs-fMRI) to describe the abnormal FC in individuals with ASDs (Kennedy and Courchesne, 2008; Assaf et al., 2010; Rudie et al., 2012; Di Martino et al., 2014). An increasing body of evidence suggests that rs-fMRI data are dynamic in essence (Hutchison et al., 2013; Allen et al., 2014), and static functional connectivity (SFC) does not clearly show the changes that occur over a short period of time during the scan (Chang and Glover, 2010). It has been proposed that quantifying changes in FC metrics over time may provide greater insight into fundamental properties of brain networks. Recently, researchers have conducted more dynamic functional connectivity (DFC) studies (Liu et al., 2017; Preti et al., 2017; Du et al., 2018). Briefly, the BOLD signals in rs-fMRI time series were divided into some overlapping intervals, and a functional correlation matrix was derived for each of these intervals. It was possible to track the ongoing changes in FC between brain regions over time (Betzel et al., 2016; Shakil et al., 2016).

There are a few studies investigating DFC in individuals with ASDs. For example, Li et al. (2020) revealed higher DFC between the posterior cingulate cortex (PCC) and middle temporal pole in patients with ASDs than in typical controls (TCs). Chen et al. (2017) demonstrated increased DFC in the medial superior frontal gyrus and temporal pole in patients with ASDs. Harlalka et al. (2019) also observed significantly higher DFC between the attention network (AN) and the default mode network (DMN) in patients with ASDs than in TCs. However, these previous studies only performed within or between network DFC analysis, which does not provide comprehensive information on DFC changes in ASDs. Specifically, DFC analysis within a given network only provides a series of relationships between a given region and all other voxels within its network, instead of the full pattern of whole-brain dynamic connections. Likewise, DFC analysis between network only reveals patterns of connectivity between these networks without taking into account internal FC.

Recently, several studies found correlations between DFC and symptom severity, but in the opposite direction. He et al. (2018) found that decreased DFC between the PCC and right precentral gyrus was negatively associated with social motivation scores. However, Chen et al. (2017) found that greater DFC was positively related to Autism Diagnostic Observation Schedule (ADOS) total score in patients with ASDs. Li et al. (2020) also found that the increased DFC between the PCC and pars opercularis of the inferior frontal gyrus was positively associated with Social Responsiveness Scale (SRS) total raw scores, social awareness and cognition scores. Further exploration of the relationship between DFC and symptom severity may provide more insights into the pathophysiological mechanisms of ASDs.

Although the recent studies have found atypical DFC in patients with ASDs, questions remain about the genetic mechanisms of higher DFC. Barber et al. (2021) examined the heritability of rs-fMRI data of healthy young adults from the Human Connectome Project and found that heritability was moderate and tended to be higher for DFC than for SFC. Epidemiological studies have shown that patients with ASDs have high heritability, twin studies evaluating the heritability of ASDs showed high concordance rates (up to 90%) in monozygotic twins (Freitag, 2007), and common genetic variations account for approximately 50% of the genetic risk for ASDs (Gaugler et al., 2014). In the past few years, transcription-neuroimaging association analyses have emerged as a popular and powerful strategy for investigating the molecular basis of brain imaging phenotypes (Fornito et al., 2019). To the best of our knowledge, there have been no studies using such approach to identify genes related to alterations of brain DFC patterns in patients with ASDs.

**FIGURE 1:**
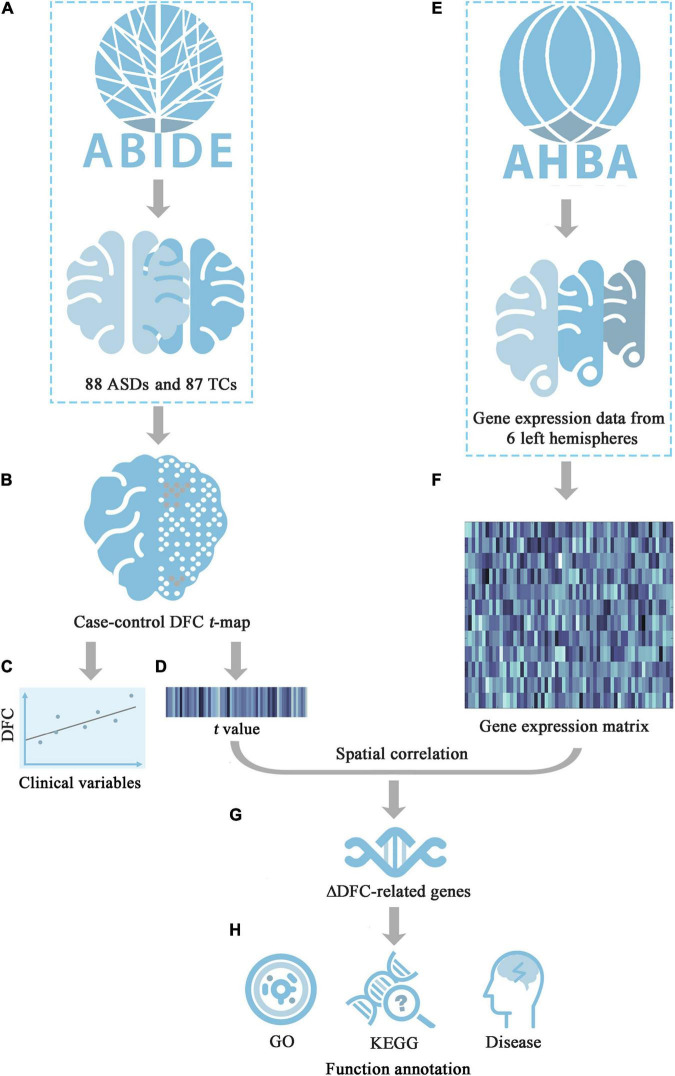
The workflow diagram of this study. **(A)** Download rs-fMRI data of ASDs and TCs groups from ABIDE II; **(B)** Calculate case-control DFC *t*-map; **(C)** Investigate the relationship between the DFC values in areas with significant group differences and symptom severity; **(D)** Extract the mean *t* value of each tissue sample; **(E)** Obtain the whole-genomic transcriptomic profiles in tissue samples from AHBA; **(F)** Generate the sample-wise gene expression matrix of six donors; **(G)** Identify ΔDFC-related genes by calculating cross-sample spatial correlation between gene expression and ΔDFC; **(H)** Functional annotations for ΔDFC-related genes, including GO, KEGG and disease enrichment analysis. ABIDE, Autism Brain Imaging Data Exchange; AHBA, Allen Human Brain Atlas; DFC, dynamic functional connectivity; GO, gene ontology; KEGG, Kyoto encyclopedia of genes and genomes; *t*, *t*-statistic.

In the current study, rs-fMRI data from Autism Brain Imaging Data Exchange II (ABIDE II) were used, and 29 core seeds of 10 classic functional networks were selected to perform sliding-window seed-to-whole-brain DFC analyses in a comprehensive manner. Moreover, the relationships between significant DFC changes and symptom severity were further explored in patients with ASDs. Furthermore, transcription-neuroimaging association analyses were conducted to explore the molecular mechanisms of the DFC alterations in patients with ASDs by leveraging the Allen Human Brain Atlas (AHBA) database. On the basis of the findings of the previous studies, we hypothesized that: (1) patients with ASDs have significantly increased DFC; (2) there are positive relationship between DFC alterations and severity of symptoms; and (3) there are correlations between DFC alterations and gene expression. An organized workflow diagram of our study is summarized in Figure 1.

## Materials and Methods

### Participants

The ASDs patient and TCs data used in this study were downloaded from the ABIDE II project (Di Martino et al., 2014, 2017), and all experimental procedures were approved by the local Institutional Review Board. The subject inclusion criteria were as follows: (1) male only, as the prevalence of ASDs has a strong male bias (Pisula and Porebowicz-Dorsmann, 2017); (2) strong right-handedness; (3) TR = 2 s, for consistency in the temporal scale (Li et al., 2020); (4) younger 18 years old, due to the greater effects of intervention on brain plasticity in children and adolescents (Dawson, 2008; Van Hecke et al., 2015); (5) subjects with Full Scale Intelligence Quotient (FIQ) within 2 standard deviation (SD) of overall ABIDE sample mean; (6) subjects with mean framewise displacement (FD) (Power et al., 2012) not exceeding 2 SD above the sample mean; (7) availability of both structural and functional images that provide complete whole-brain coverage with successful segmentation, good registration and good image quality; and (8) sites with at least 10 subjects in each group after meeting the above criteria (Di Martino et al., 2014). Finally, 88 patients with ASDs and 87 TCs were included in our study; the number of the subjects excluded due to each of the exclusion criteria is shown in Supplementary Figure 1. The detailed demographic information of each site (Supplementary Table 1) and MRI acquisition parameters are summarized in the Supplementary Materials. For more information, see http://fcon_1000.projects.nitrc.org/indi/abide/.

The ADOS is a tool that can be used by clinical doctor to perform a standardized clinical observation of a child (Lord et al., 2012), and comprises two behavioral domains: restricted and repetitive behaviors and social affect. The SRS is a parent-report quantitative assessment scale (Bruni, 2014), and designed to evaluate children’s social deficits. SRS provides a total score and separate scores for five subdomains, including social awareness, social cognition, social communication, social motivation, and autistic mannerisms. Separate scores of each domain would provide a clearer picture of ASDs dimensions. These tools offer distinct information from different sources and perspectives (Duvekot et al., 2015), and the complementary information contributed to acquire a comprehensive view of the characteristics of ASDs. The ADOS and SRS scores can be used to assess the severity of symptoms related to ASDs, and higher scores indicate more severe ASD symptoms (Plitt et al., 2015; Chen et al., 2019; Li et al., 2020).

### Magnetic Resonance Imaging Data Pre-processing

Both structural and functional images of all the subjects were examined independently by two researchers, and all images were reoriented to the anterior--posterior commissure line. Functional images were pre-processed using the Data Processing and Analysis for Brain Imaging (DPABI)^1^ toolbox (Yan et al., 2016). Specifically, the first five volumes of each subject were removed to allow the signal to reach equilibrium. Slice timing and realignment were then performed to correct the temporal differences between slices and head motion. The mean FD was calculated based on head motion parameters (Van Dijk et al., 2012), and subjects with mean FD > 0.5 were excluded (no subjects were excluded in this step). Next, individual structural images were co-registered to the mean motion-corrected functional images, the transformed structural images were segmented into gray matter (GM), white matter (WM), and cerebrospinal fluid (CSF), and the motion-corrected functional images were normalized spatially to the standard Montreal Neurological Institute (MNI) space using the normalization parameters estimated by the Diffeomorphic Anatomical Registration Through Exponentiated Lie algebra (DARTEL) (Ashburner, 2007) tool and resampled to 3 mm cubic voxels. Subsequently, nuisance covariates [including linear trend, Friston-24 head motion parameters (Friston et al., 1996) and mean signals from WM and CSF] were regressed out, and temporal bandpass filtering (0.01–0.08 Hz) was applied. Finally, the functional images were spatially smoothed with an 8 mm full-width at half-maximum Gaussian kernel.

### Resting State Network Selection and Dynamic Functional Connectivity Calculation

Based on prior studies (Damoiseaux et al., 2006; Mantini et al., 2007; Power et al., 2010; van den Heuvel and Hulshoff Pol, 2010; Zuo et al., 2010; Allen et al., 2011), 29 core seeds within 10 classic brain networks were selected as regions of interest (ROIs) in this study. Specifically, spherical regions with radius of 6 mm centered at the MNI coordinates served as the ROIs. These regions were as follows: (1) the AN: bilateral superior temporal gyrus (STG) (Albouy et al., 2013); (2) the central executive network (CEN): bilateral dorsolateral prefrontal cortex (DLPFC) and bilateral posterior parietal cortex (Denkova et al., 2019); (3) the dorsal attention network (DAN): bilateral frontal eye field (FEF) and bilateral intraparietal sulcus (IPS) (McCarthy et al., 2013); (4) the DMN: medial prefrontal cortex and PCC (Fox et al., 2005); (5) the dorsal visual network: bilateral superior occipital gyrus (Shen et al., 2019); (6) the primary visual network: calcarine fissure (Shen et al., 2019); (7) the sensorimotor network: bilateral precentral gyrus, bilateral postcentral gyrus and bilateral supplementary motor area (Behroozmand et al., 2015); (8) the salience network: dorsal anterior cingulate cortex and bilateral frontoinsular cortex (Denkova et al., 2019); (9) the ventral attention network: bilateral orbitofrontal cortex and bilateral temporoparietal junction (Majerus et al., 2012); and (10) the ventral visual network (VVN): calcarine gyri (Shen et al., 2019). The detailed MNI coordinates and the spatial distribution of these ROIs are shown in Supplementary Table 2 and Supplementary Figure 2, respectively.

To calculate the whole-brain resting-state DFC map of each ROI, a widely used sliding-window approach was adopted. First, as suggested by previous studies (Li et al., 2019; Christiaen et al., 2020), a window length of 50 TRs (100 s) and a step size of 1 TR (2 s) were employed to obtain windowed time series. Second, in each window, the whole-brain DFC map for each ROI was created for each subject by calculating Pearson’s correlation coefficient between the mean time series of all voxels in the ROI and the time series from all other brain voxels in the GM. Third, Fisher’s *r*-to-*z* transformation was applied for all DFC maps to improve the normality of the correlation distribution. Finally, the SD map across time windows was calculated in each subject to characterize the changes in individual ROI-to-whole-brain DFC, which is a commonly used metric in previous DFC studies (Falahpour et al., 2016; Kaiser et al., 2016; Harlalka et al., 2019; Xue et al., 2020).

A ComBat approach^2^, which can remove intersite variation, preserve biological variability, and is robust to small sample size data, was utilized to harmonize individual DFC maps from the five independent datasets (Fortin et al., 2017). Subsequently, a voxel-wise general linear model was used to compare the DFC maps of each ROI between the ASDs and TCs groups while controlling for age, FIQ and mean FD. The Gaussian random-field (GRF) method was used to correct for multiple comparisons of the resulting statistical map with significant thresholds of voxel level *p* < 0.001 and cluster level *p* < 0.0017 (0.05/29 ROIs). Furthermore, linear regression analyses were conducted to assess the relationship between the DFC from significant clusters of between-group comparison and symptom severity in patients with ASDs with age, FIQ, and mean FD as nuisance covariates.

### Gene Expression Data Processing

Gene expression data were obtained from the AHBA. The AHBA comprises the normalized expression data of 20737 genes represented by 58692 probes taken from 3702 brain tissue samples from six donors (one female and five males, aged 24–57 years) (Hawrylycz et al., 2012). According to the pipeline (Arnatkeviciute et al., 2019), the gene expression data preprocessing steps included (1) gene information reannotation, (2) data filtering, (3) probe selection, (4) sample assignment, (5) gene filtering, and (6) accounting for spatial effects. Given that all donors provided tissue samples from the left hemisphere but only two donors provided samples from the right hemisphere, tissue samples from the left hemisphere were used in the following analyses. In addition, consistent with previous studies (Kang et al., 2011; Fan et al., 2016), we restricted our analyses to the cerebral cortex due to the substantial differences in the gene expression patterns of the cerebral cortex, subcortex, and cerebellum. Finally, we obtained a normalized gene expression matrix of 1285 × 10185 (sample × gene). The detailed pre-processing steps are described in Supplementary Material, and the process of brain tissue samples selection is presented in Supplementary Figure 3.

### Transcription-Neuroimaging Association Analysis

For each of the *t*-maps that survived from multiple comparisons, the mean *t* value of the spherical region with a 6 mm radius centered at the MNI coordinate of each tissue sample was extracted based on the uncorrected case-control DFC *t*-maps, and the mean *t* value was defined as the ΔDFC of the sample. Then, a gene-wise across-sample spatial correlation was performed to explore the correlation between gene expression and ΔDFC in patients with ASDs. Multiple comparison correction was performed using the Benjamini-Hochberg false discovery rate (BH-FDR) method (*q* < 0.05), and the surviving genes were defined as ΔDFC-related genes.

Functional annotations of the identified ΔDFC-related genes of each ROI were created with the WEB-based WebGestalt Toolkit (Zhang et al., 2005)^3^, which includes Gene Ontology (GO) (enriching genes for specific biological processes, cellular components and molecular functions) (Ashburner et al., 2000) and Kyoto Encyclopedia of Genes and Genomes (KEGG) (identifying genes associated with specific biological pathways) databases (Kanehisa et al., 2004). In addition, DisGeNET, which contains one of the largest publicly available datasets of genes and variants associated with human diseases (Pinero et al., 2017), was used to enrich the ΔDFC-related genes for specific neurodegenerative and neuropsychiatric diseases. The intersection of enriched pathways that the identified ΔDFC-related genes of each ROI for were considered to be stably related to the genetic mechanisms of abnormal DFC patterns in patients with ASDs.

### Validation Analyses

To verify the robustness of our main findings, we performed the following three experiments.

First, we performed validation analyses for different window sizes (30 TRs and 70 TRs). We investigated whether the results (including those of between-group DFC comparisons, DFC-symptom severity associations and ΔDFC-related gene identification) of 50 TRs could be reproducible in other window sizes.

Second, we calculated the whole-brain resting-state SFC map of each ROI to compare with the DFC results. For each subject, the whole-brain SFC map for each ROI was created by calculating Pearson’s correlation coefficient between the mean time series of all voxels in the ROI and the time series from all other brain voxels in the GM. Then, Fisher’s *r*-to-*z* transformation was applied for all SFC maps to improve the normality of the correlation distribution. The subsequent procedures were the same as those mentioned above in the DFC analysis.

Third, although the mean FD was regressed out using general linear model when examining the DFC changes, we could only control the linear effect of the head motion. To further investigate whether the increased DFC is linked to the higher head motion in ASDs, a median split based on FD in both ASD and TC groups was performed and DFC were compared in the subgroups of ASD and TC.

## Results

### Demographic Information and Clinical Characteristics

In this study, 88 patients with ASDs and 87 TCs from 5 research sites met the inclusion criteria. In the ASDs group, 61 subjects had ADOS-2 scores and 69 subjects had SRS scores; In the TCs group, no subject had ADOS-2 scores and 71 subjects had SRS scores. The two groups were matched for age and FIQ. There were significant differences in mean FD, ADOS, and SRS total score and all subscale scores. The detailed demographic and clinical information of the participants is displayed in Table 1.

**TABLE 1:** Demographic and clinical information of the participants.

Variables	ASDs (*n* = 88) (Mean ± SD)	TCs (*n* = 87) (Mean ± SD)	*p* value
Age (years)	(5.43–17.93) 11.30 ± 2.68	(5.90–17.60) 11.30 ± 2.65	0.987
FIQ	112.43 ± 13.85	114.69 ± 12.50	0.259
Mean FD	0.115 ± 0.073	0.090 ± 0.054	0.012
ADOS-2 calibrated severity total score	6.87 ± 2.15 (*n* = 61)	–	–
SRS total score	89.00 ± 29.51 (*n* = 69)	19.80 ± 13.55 (*n* = 71)	<0.001
SRS subscale score (raw)			
Awareness	11.71 ± 3.94	4.30 ± 2.72	<0.001
Cognition	15.90 ± 5.9	2.66 ± 2.56	<0.001
Communication	30.23 ± 11.01	6.37 ± 5.32	<0.001
Motivation	14.61 ± 5.77	4.14 ± 3.26	<0.001
Mannerisms	16.55 ± 7.39	2.34 ± 3.00	<0.001

*ADOS, Autism Diagnostic Observation Schedule; ASDs, autism spectrum disorders; FD, framewise displacement; FIQ, Full-scale Intelligence Quotient; SCQ, Social Communication Questionnaire; SD, standard deviation; SRS, Social Responsiveness Scale; TCs, typical controls.*

*p values were obtained by two-sample t-tests; –, not available.*

### Case-Control Dynamic Functional Connectivity Differences

**FIGURE 2:**
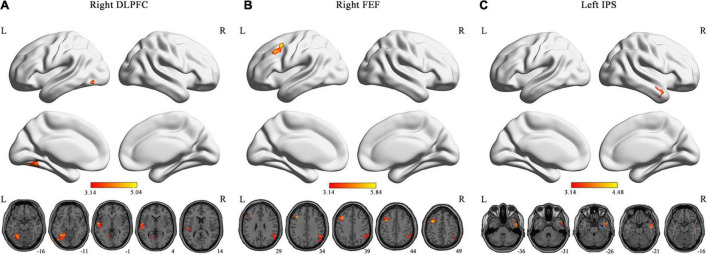
Brain regions with significant DFC alterations. The brain regions exhibited significant DFC alterations (GRF-corrected *p* < 0.0017) with the right DLPFC **(A)** of CEN, and the right FEF **(B)** and left IPS **(C)** of DAN in the patients with ASDs, respectively. The color bar represents *t*-statistic. DLPFC, dorsolateral prefrontal cortex; FEF, frontal eye field; IPS, intraparietal sulcus; L, left; R, right.

We found that patients with ASDs exhibited significantly increased DFC between the right DLPFC of the CEN and the left fusiform gyrus (FFG)/lingual gyrus (LG), and between the right DLPFC of the CEN and the left STG; significantly increased DFC between the right FEF of the DAN and the left middle frontal gyrus (MFG), and between the right FEF of the DAN and the right angular gyrus (AG); and significantly increased DFC between the left IPS of the DAN and the right middle temporal gyrus (MTG) compared with the TCs [GRF corrected, voxel level *p* < 0.001 and cluster level *p* < 0.0017 (0.05/29 ROIs)] (Figure 2). There was no significantly decreased DFC in the patients with ASDs compared with the TCs. Detailed information on the brain regions with significant ΔDFC in the patients with ASDs is presented in Table 2. The uncorrected case-control *t*-maps for the 29 core seeds in 10 classic resting state networks shown in Supplementary Figures 4–13.

**TABLE 2:** Detailed information on the brain regions with significant ΔDFC in the patients with ASDs (GRF-corrected *p* < 0.0017).

ROIs	Region	MNI coordinates (x, y, z)	Cluster size	Peak *T*
R DLPFC	L FFG/LG	−36, −57, −12	286	4.604
	L STG	−63, −27, 6	235	5.041
R FEF	R AG	60, −57, 27	168	4.166
	L MFG	−36, 21, 36	178	5.839
L IPS	R MTG	54, −6, −21	146	4.476

*AG, angular gyrus; DLPFC, dorsolateral prefrontal cortex; FEF, frontal eye field; FFG, fusiform gyrus; IPS, intraparietal sulcus; L, left; LG, lingual gyrus; MFG, middle frontal gyrus; MNI, Montreal Neurological Institute; MTG, middle temporal gyrus; R, right; ROIs, regions of interest; STG, superior temporal gyrus; T, t-statistic.*

### Correlations Between ΔDFC and Symptom Severity

**FIGURE 3:**
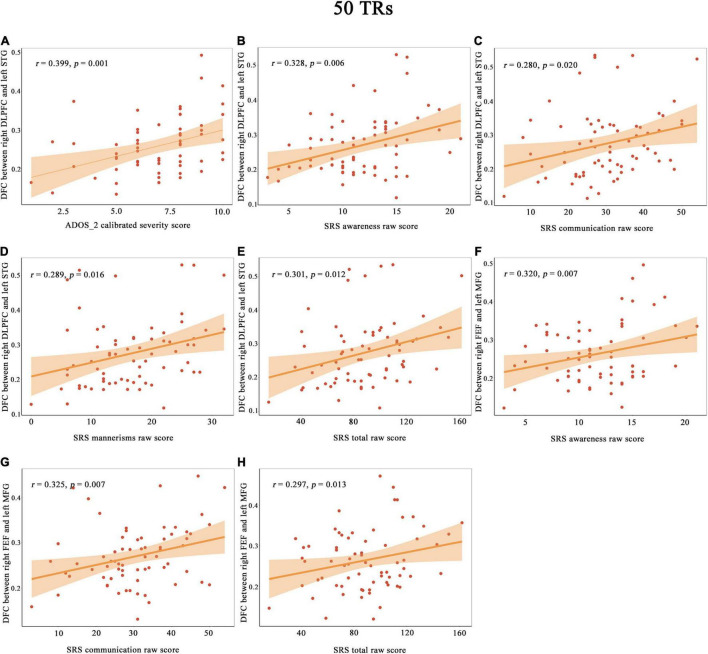
The correlation between the DFC of the right DLPFC with the left STG and with ADOS_2 calibrated severity score **(A)**, SRS awareness raw score **(B)**, SRS communication raw score **(C)**, SRS mannerisms raw score **(D)** and SRS total raw score **(E)**. The correlation between the DFC of the right FEF with the left MFG and with SRS awareness raw score **(F)**, SRS communication raw score **(G)** and SRS total raw score **(H)**. Shades represent the 95% confidence intervals. ADOS, Autism Diagnostic Observation Schedule; DLPFC, dorsolateral prefrontal cortex; FEF, frontal eye field; FFG, fusiform gyrus; LG, lingual gyrus; MFG, middle frontal gyrus; SRS, Social Responsiveness Scale; STG, superior temporal gyrus; *p*, uncorrected *p* value.

As illustrated in Figure 3, the DFC of the right DLPFC with left STG were positively correlated with ADOS-2 calibrated severity total score in the patients with ASDs. In addition, the DFC of the right DLPFC with the left STG was positively correlated with the SRS social awareness score, social communication score, autism mannerisms score and total scores in the patients with ASDs. The DFC of the right FEF with the left MFG was positively correlated with the SRS social awareness score, social communication score and total scores in the patients with ASDs.

### Transcription-Neuroimaging Association Analysis

Gene-wise across-sample spatial correlation analysis was performed between ΔDFC [three significant *t*-maps, with the three seeds (the right DLPFC, right FEF and left IPS)] and gene expression. After multiple comparison correction (BH-FDR *q* < 0.05), 6803, 2722, and 1217 genes survived, respectively. Subsequently, gene functional annotation analysis was performed on the ΔDFC-related genes in the three groups.

**FIGURE 4:**
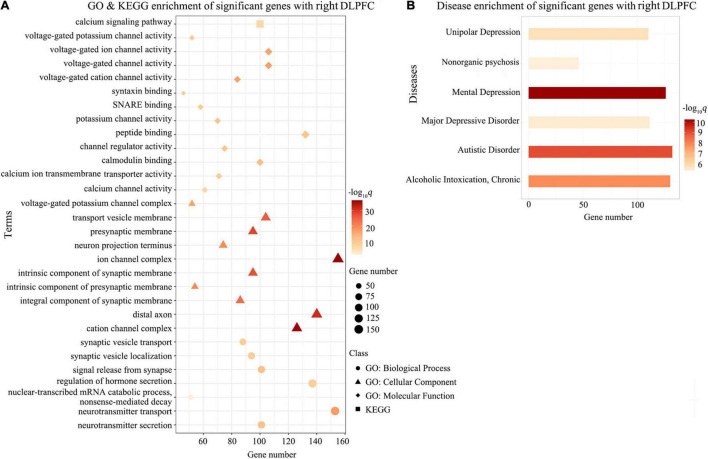
Enrichment analysis for ΔDFC-related genes with the right DLPFC in CEN. The GO and KEGG enrichment analysis **(A)** and the disease enrichment analysis **(B)** for ΔDFC-related genes with the right DLPFC in CEN in the patients with ASDs. The color bars represent –log_10_*q* with BH-FDR correction, and the size of circles (GO: biological processes), triangles (GO: cellular component), rhombus (GO: molecular functions), and squares (KEGG terms) represents the overlapping gene number. DLPFC, dorsolateral prefrontal cortex; GO, Gene Ontology; KEGG, Kyoto Encyclopedia of Genes and Genomes.

**FIGURE 5:**
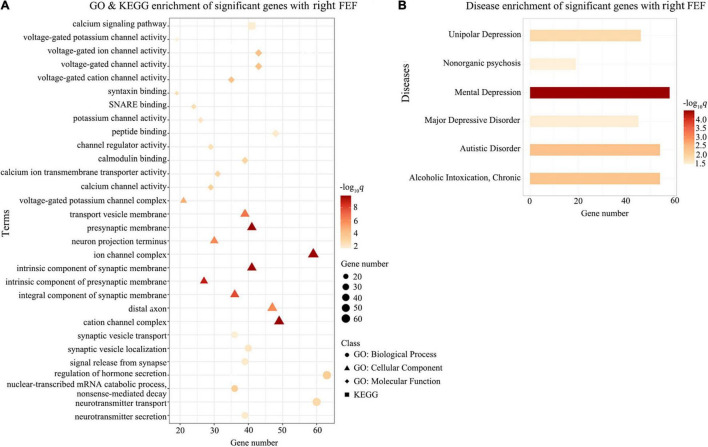
Enrichment analysis for ΔDFC-related genes with the right FEF in DAN. The GO and KEGG enrichment analysis **(A)** and the disease enrichment analysis **(B)** for ΔDFC-related genes with the right FEF in DAN in the patients with ASDs. The color bars represent –log_10_*q* with BH-FDR correction, and the size of circles (GO: biological processes), triangles (GO: cellular component), rhombus (GO: molecular functions), and squares (KEGG terms) represents the overlapping gene number. FEF, frontal eye field; GO, Gene Ontology; KEGG, Kyoto Encyclopedia of Genes and Genomes.

**FIGURE 6:**
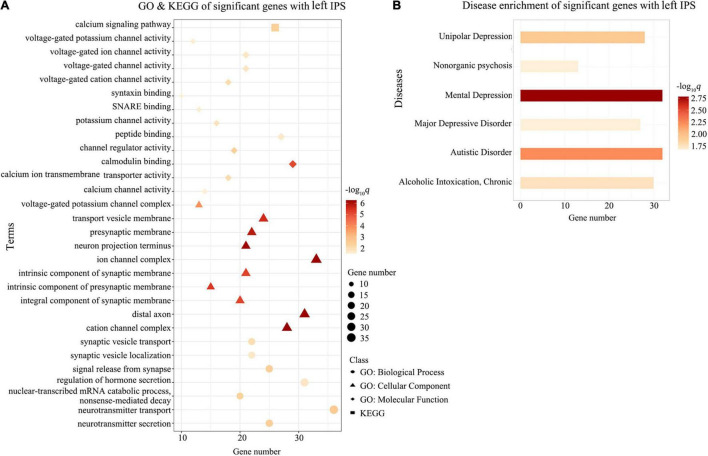
Enrichment analysis for ΔDFC-related genes with the left IPS in DAN. The GO and KEGG enrichment analysis **(A)** and the disease enrichment analysis **(B)** for ΔDFC-related genes with the left IPS in DAN in the patients with ASDs. The color bars represent –log_10_*q* with BH-FDR correction, and the size of circles (GO: biological processes), triangles (GO: cellular component), rhombus (GO: molecular functions), and squares (KEGG terms) represents the overlapping gene number. GO, Gene Ontology; IPS, intraparietal sulcus; KEGG, Kyoto Encyclopedia of Genes and Genomes.

Gene Ontology enrichment analysis showed that the ΔDFC-related genes were significantly enriched for the biological process of neurotransmitter secretion, neurotransmitter transport, the cellular component of ion channel complexes and synaptic membranes, and molecular function of voltage-gated ion channel activity. KEGG enrichment analysis revealed that the ΔDFC-related genes were significantly enriched in the calcium signaling pathway. In the disease-related enrichment analyses, the ΔDFC-related genes were significantly enriched for autistic disorder, chronic alcoholic intoxication, several disorders related to depression and non-organic psychosis. Please see the detailed results of enrichment analysis in Figures 4–6.

### Validation Results

Our results showed high reproducibility across the different window sizes. We found that the brain regions with significant group differences (50 TRs) in DFC remained significantly different between the ASDs and TCs groups for both 30 TR and 70 TR windows. Moreover, we observed highly similar results in the correlation analyses of the DFC values in areas with significant group differences and symptom severity across the three window sizes. Moreover, the significantly enriched pathways (50 TRs) were largely reproducible for 30 TR and 70 TR windows. Please see the detailed results in Supplementary Figures 14–17. We found no significant difference in SFC in the patients with ASDs compared with the TCs. In addition, the direction of effect (higher DFC in ASDs) in these comparisons of subgroups with a median split based on FD was consistent with our main results (Supplementary Table 3 and Supplementary Figure 18).

## Discussion

In this study, we assessed the DFC alterations among the 29 core seeds of 10 classic resting-state networks and the whole brain in patients with ASDs compared with TCs and calculated the correlation between the DFC values in areas with significant group differences and symptom severity in patients with ASDs. In patients with ASDs, we observed significantly increased DFC between the right DLPFC (the core seed of the CEN) and the left FFG/LG, between the right DLPFC and the left STG; between the right FEF (the core seed of the DAN) and the left MFG, between the right FEF and the right AG, and between the left IPS (the core seed of the DAN) and the right MTG.

High DFC could lead to the instability of information transfer within and between networks (Kung et al., 2019). The DLPFC is a core region in the CEN and is responsible for subjective feelings, self-awareness, emotion regulation, working memory, executive functions and the judgment and decision making under goal-directed behavior (Bunge et al., 2001; Craig, 2002; Bressler and Menon, 2010). The FFG and LG are key regions in the VVN that are associated with visual item identification, such as face recognition, memory for visual item identity and planning a response to potentially threatening stimuli (Slotnick and Schacter, 2006; Jonas et al., 2015; Li et al., 2016). The increased DFC between the CEN and VVN may be related to the loss of emotional regulation and social interaction impairment in patients with ASDs (Li et al., 2016; Bi et al., 2018). The MFG is also a core region in the CEN and is mainly responsible for integrating and processing information (Richeson et al., 2003; Bi et al., 2018). Previous studies have suggested that individuals with ASDs are unable to integrate and process information; therefore, they cannot communicate normally with others (Bi et al., 2018). We speculated that the increased DFC between the CEN and DAN may be associated with communication defects in patients with ASDs.

Frontal eye field and IPS are core regions in the DAN that are involved in mediating many higher-order cognitive tasks and supporting top-down attention to visual, auditory and somatosensory inputs (Braga et al., 2013; McCarthy et al., 2013; Xia et al., 2015; Rohr et al., 2017). The AG is an important part of the DMN, which is implicated in social cognition and affective regulation associated with empathic responses (Li et al., 2013, 2016; Vatansever et al., 2017). The increased DFC between the DAN and DMN might lead to deficits in attention switching and cognitive function in patients with ASDs (Jia et al., 2020; Meeker et al., 2021). The STG and MTG are crucial regions in the AN that are associated with auditory language, visual language and emotion (Li et al., 2013; Orlov et al., 2018; Luan et al., 2019). The increased DFC between the CEN/DAN and AN may be related to social interaction impairment in patients with ASDs. Similar to our findings, some studies observed no significant difference in SFC between the ASDs and TCs groups (Chen et al., 2015; Li et al., 2020). No significant difference has been observed in the SFC analyses, which is partially because DFC can capture the time-varying properties and seems to be more sensitive than SFC (Li et al., 2020).

In the correlation analyses between the DFC and symptom severity in patients with ASDs, we observed positive correlations between the DFC of the right DLPFC (core seed of the CEN) and the left STG (core seed of the AN) and ADOS-2 calibrated severity total score. ASDs is characterized by socio-communicational deficits and restricted and repetitive behaviors. Higher severity total score indicates more severe ASDs symptoms. Functional alterations of CEN have previously been reported in ASDs (Perez Velazquez et al., 2009). Alterations of the AN in ASDs are associated with the severity of autistic core symptom (Watanabe and Rees, 2016). The AN is mainly involved with lower-level perception (Power et al., 2011). The CEN controls attention, integrates the information processed in the other networks and plays a central role in various cognitive functions (Chadick and Gazzaley, 2011; Zanto and Gazzaley, 2013). The higher DFC between the DLPFC and STG could cause unstable transmission and impaired coupling between these brain regions. In addition, underconnectivity theory proposes that both the two core symptoms of ASDs are associated with impairment of integration of global information (Kana et al., 2011; Just et al., 2012). Therefore, the higher DFC between the DLPFC and STG observed in our study might cause the more severe ASDs symptoms.

Additionally, we observed a positive correlation between the DFC of the right DLPFC with the left STG and with the patients’ SRS social awareness score, social communication score, autism mannerisms score and total score. We also revealed a positive correlation between the DFC of the right FEF with the left MFG and with the patients’ SRS social awareness score, social communication score and total score. Social awareness contributes to one’s ability to recognize and understand the thinking or feeling of others (Li et al., 2020), social communication is one’s capability to respond appropriately to what others interpret (He et al., 2018), and autism mannerisms describe a collection of stereotypical behaviors or restricted interests (Plitt et al., 2015). The higher DFC between the DLPFC (core seed of the CEN) and STG (core seed of the AN) indicated unstable information transmission (Chen et al., 2017) and might influence auditory attention, semantic fluency, the learning of social cues and the integration of appropriate social responses. Furthermore, we found that the DFC between the right FEF (core seed of the DAN) and the left MFG (key region of the CEN) was positively correlated with the SRS social awareness score, social communication score and total score. Both the CEN and DAN are involved in cognitive regulation and may be related to social awareness. Individuals with ASDs cannot communicate normally with others due to deficits in integrating and processing information, and the MFG is mainly responsible for integrating and processing information (Richeson et al., 2003; Bi et al., 2018). This important detail may explain the positive correlation among the DFC of the right FEF-left MFG, the patient’s SRS social awareness score, and the patient’s communication score.

Overall, the ΔDFC-related genes were mainly enriched for voltage-gated ion channels, especially calcium and potassium channels, synaptic membranes and the related processes involved in the release and transmission of neurotransmitters. Voltage-gated ion channels are important mediators of physiological functions in the central nervous system. Activation of these channels influences neurotransmitter release, neuronal excitability, gene transcription, and plasticity. Ion channels, especially polymorphisms in calcium and potassium channels, are related to the pathogenesis of ASDs (Imbrici et al., 2013). Moreover, calcium signaling is ubiquitously involved in the process of neuronal excitability, neurotransmitter release and cell secretion (Schmunk et al., 2017). Genetic mutations related to the calcium signaling pathway can elevate the risk of developing ASDs (Cross-Disorder Group of the Psychiatric Genomics Consortium, 2013). Membrane proteins are significant components of the proteins in cells and play a key role in synaptic transmission; also, disruption of synaptic membrane components may influence synaptic signaling transmission (Wang et al., 2021). Abnormalities in synapse formation, which contribute to functional and cognitive impairments, play a vital role in the pathological mechanism of ASDs (Bourgeron, 2007). Neurotransmitters can transmit nerve impulses from neurons to other cells. Neurotransmitter transport dysfunction can affect the transmission and absorption of neurotransmitters (Bhat et al., 2021), and abnormalities in the balance between excitatory and inhibitory neurotransmission may be connected with the etiology of ASDs (Horder et al., 2018).

In the disease-related enrichment analyses, the ΔDFC-related genes were enriched for autistic disorder, chronic alcoholic intoxication, several disorders related to depression and non-organic psychosis. Upon initiation of alcohol use, individuals with ASDs were at higher risk for developing alcohol dependence (Sizoo et al., 2010). Studies have shown an increased risk for depression symptoms in children and adolescents with ASDs who had various IQs (Strang et al., 2012). Individuals with ASDs are associated with a substantially increased risk for non-affective psychotic disorder (NAPD) and bipolar disorder; notably, non-organic psychosis was the most commonly diagnosed subtype of NAPD among individuals with ASDs (Selten et al., 2015). Deficits in social interactions and emotion regulation in individuals with ASDs may be associated with elevated rates of psychiatric comorbidity (Pouw et al., 2013; Charlton et al., 2020).

Several limitations should be considered in this study. First, compared with single-site studies, the use of multicenter public imaging data in this study may have involved some issues associated with scanner differences and inconsistency assessments. Second, head motion was a confounding factor in DFC analyses. To reduce this effect, we carried out a series of procedures, including regressing 24 head motion parameters in the data pre-processing, and controlling for mean FD in group-comparisons (Yan et al., 2013). However, the effect of motion cannot be completely ruled out. In the future, it would be better to recruit subjects with matched head motion to replicate the findings. Third, although many dynamic metrics could be used to investigate DFC, we employed SD of FC values across time windows to characterize DFC because it was difficult to calculate other dynamic metrics due to the highly computational burden. Further study is needed to calculate other dynamic metrics of seed-based voxel-level DFC by using high-performance computing systems. Fourth, the results about the genes expressed in autistic disorder, chronic alcoholic intoxication and disorders related to depression were obtained from the disease enrichment analysis, the detailed information about these conditions of the autism patients included in our study was not provided by ABIDE database. Fifth, we did not include female subjects or investigate the sex-related effects in this study due to the highly male-biased sex ratios in ABIDE. Sixth, the weaker associations between DFC and symptom severity in patients with ASDs may be related to the relatively small sample size. Seventh, the average *t*-statistics were extracted around the location of AHBA tissue samples; however, the uneven spatial distribution of tissue samples in the AHBA may influence our results. Finally, in our study, the imaging and gene expression data were not derived from the same subjects. However, many studies have confirmed that genes involved in the regulation of transcription and development across human populations are highly conserved (Bejerano et al., 2004; Woolfe et al., 2005); therefore, our results of transcription-neuroimaging association studies should be reliable.

In conclusion, the patients with ASDs showed increased DFC in brain areas related to attentional and cognitive regulation, which were associated with symptom severity. Transcription-neuroimaging association analyses identified ΔDFC-related genes, especially those involved in processes associated with synaptic signaling, voltage-gated ion channels, neurotransmitter secretion and transport. These findings provide more insight into the polygenetic and multipathway molecular mechanisms of functional impairments in patients with ASDs.

## Data Availability Statement

The original contributions presented in the study are included in the article/Supplementary Material, further inquiries can be directed to the corresponding author/s.

## Ethics Statement

Ethical review and approval were not required for the current study in accordance with the local legislation and institutional requirements. All research procedures and ethical guidelines pertaining to the existing datasets were approved by the local Institutional Review Board of each participating institution (Di Martino et al., 2014, 2017). Written informed consent to participate in these previous studies was provided by the participants’ legal guardian/next of kin.

## Author Contributions

JW, FL, and ZY designed the research and provided the guidance for the study. LM, TY, and WL processed the images and analyzed the data. LG, DZ, and ZW visualized the results. ZL, KX, and YW checked the data quality. JL and WM modified the language. LM wrote the initial draft. All authors read and approved the final manuscript.

## Conflict of Interest

The authors declare that the research was conducted in the absence of any commercial or financial relationships that could be construed as a potential conflict of interest.

## Publisher’s Note

All claims expressed in this article are solely those of the authors and do not necessarily represent those of their affiliated organizations, or those of the publisher, the editors and the reviewers. Any product that may be evaluated in this article, or claim that may be made by its manufacturer, is not guaranteed or endorsed by the publisher.

## Footnotes

1
http://rfmri.org/dpabi

2
https://github.com/Jfortin1/ComBatHarmonization

3
http://www.webgestalt.org/
