# Supplementary material for: Dynamic Functional Connectivity Alterations and Their Associated Gene Expression Pattern in Autism Spectrum Disorders
Source: Front Neurosci. 2022 Jan 10;15:794151. doi: 10.3389/fnins.2021.794151 (PMC8784878; doi:10.3389/fnins.2021.794151)
Supplement: Supplementary file 1 [file Data_Sheet_1.pdf]

Supplementary Material

1 Supplementary Content

1.1 Image Acquisition

Georgetown University (GU)

Functional images were acquired on a Siemens Trio 3T scanner with a T2*-sensitive gradient echo pulse sequence: 154 volumes; repetition time (TR) = 2000 ms; echo time (TE) = 30 ms; flip angle (FA) = 90°; 43 slices; voxel size = 3.0 mm × 3.0 mm × 2.5 mm; field of view (FOV) = 256 mm × 256 mm. High-resolution structural images were collected with a MPRAGE sequence: TR = 2530 ms; TE = 3.5 ms; FA = 7°; 176 slices; FOV = 256 mm × 256 mm.

NYU Langone Medical Center (NYU)

Functional images were acquired on a Siemens Allegra 3T scanner with a multi-echo planar imaging (EPI) sequence: 180 volumes; TR = 2000 ms; TE = 15 ms; FA = 90°; 33 slices; voxel size = 3 mm × 3 mm × 4 mm; FOV = 240 mm × 192 mm. High-resolution 3D T1-weighted images were acquired with a gradient echo sequence: TR = 2530 ms; TE = 3.25 ms; FA = 7°; 128 slices; FOV = 256 mm × 256 mm.

San Diego State University (SDSU)

Functional images were acquired on a GE MR750 3T scanner with an EPI pulse sequence: 180 volumes; TR = 2000 ms; TE = 30 ms; FA = 90°; 42 slices; voxel size = 3.4 mm × 3.4 mm × 3.4 mm; FOV = 220 mm × 220 mm. High-resolution structural images were collected with an FSPGR T1-weighted sequence: TR = 8.13 ms; TE = 3.17 ms; FA = 8°; 128 slices; FOV = 256 mm × 256 mm.

Stanford University (SU)

Functional images were acquired on a GE SIGNA 3T scanner with a spiral in-out pulse sequence: 180 volumes; TR = 2000 ms; TE = 30 ms; FA = 80°; 31 slices; voxel size = 3.4 mm × 3.4 mm × 3.5 mm; FOV = 220 mm × 220 mm. High-resolution structural images were collected with an ANAT T1 sequence: TR = 5.9 ms; TE = 1.8 ms; FA = 11°; 132 slices; FOV = 240 mm × 240 mm.

Trinity College Dublin (TCD)

Functional images were acquired on a Philips Intera Achieva 3T scanner with an EPI sequence: 210 volumes; TR = 2000 ms; TE = 27 ms; FA = 90°; 37 slices; voxel size = 3.5 mm × 3.5 mm × 3.5 mm; FOV = 240 mm × 240 mm. High-resolution structural images were collected with an FSPGR T1-weighted sequence: TR = 8.39 ms; TE = 3.9 ms; FA = 8°; 180 slices; FOV = 230 mm × 230 mm.

1.2 The information about how the participants were diagnosed with ASDs

GU

ASD diagnosis included DSM-IV-TR criteria applied by a trained clinician and confirmed with Autism Diagnostic Interview-Revised (ADI-R) and Autism Diagnostic Observation Schedule-Generic (ADOS-G) following the criteria established by the NICHD/NIDCD Collaborative Programs for Excellence in Autism. These criteria require that the child meet ADI-R cutoff for autism in the social domain and at least one other domain (communication and/or repetitive behaviors and

restricted interests), and meet Autism Diagnostic Observation Schedule (ADOS) cutoff (autism or ASD) for the combined social and communication score.

NYU

Inclusion as a participant with ASD required a clinician's DSM-IV-TR diagnosis of Autistic Disorder, Asperger's Disorder, or Pervasive Developmental Disorder Not-Otherwise-Specified or DSM-5 diagnosis of ASD, for those enrolled after the DSM-5 release in 2013. In such cases, clinicians reviewed the case diagnoses using both DSM-IV-TR and DSM-5 codes. Diagnosis was supported by review of available records, an ADOS, review of the participant's history, and when possible, an ADI-R.

SDSU

Clinical diagnoses were confirmed using all of the following: the Autism Diagnostic Interview-Revised, the ADOS (2nd edition), and expert clinical judgment according to DSM-5 criteria. Clinical assessments were confirmed by expert clinical neuropsychologists.

SU

Children with ASD received a diagnosis based on scores from the ADI-R and/or the ADOS administered by a research reliable clinician. Children with ASD were screened through a parent phone interview and excluded if they had any history of known genetic, psychiatric, or neurological disorders (e.g., Fragile X syndrome or Tourette's syndrome), or were currently prescribed antipsychotic medications.

TCD

Inclusion Criteria of subjects with ASD: (1) right-handed males aged 10-20 years of age; (2) meeting cutoff criteria at the ADI; (3) meeting cutoff criteria at the ADOS-G for autism and ASD; (4) meeting cutoff scores for both ADI-R and ADOS-G.

1.3 The new preprocessing pipeline for gene expression data

The gene expression data of six left hemispheres were preprocessed using a newly proposed pipeline (Arnatkeviciute et al., 2019) available in the github (https://github.com/BMHLab/AHBAprocessing). We updated the annotations for Microarray Probe Sequences by using Re-Annotator package and obtained 20232 unique genes; we excluded probes with expression measure inferior to the background in more than 50% samples; we removed genes without the corresponding RNA-seq measures; we removed probes that had correlation to RNA-seq data lower than 0.2; we selected a representative probe with the highest correlation to the RNA-seq expression data in corresponding samples. In addition, consistent with previous studies (Kang et al., 2011; Fan et al., 2016), we restricted our analyses to the cerebral cortex due to the substantial differences in the gene expression patterns of the cerebral cortex, subcortex, and cerebellum. We normalized gene expression data using the scaled robust sigmoid transform. Finally, a normalized gene expression matrix of 1285 × 10185 (sample × gene) was obtained based on these processing procedures. The detailed preprocessing parameters we selected were as follows: (1) options.ExcludeCBandBS = true; (2) options.useCUSTprobes = true; (3) options.updateProbes = 'reannotator'; (4) options.probeSelections = {'RNAseq'}; (5) options.signalThreshold = 0.5; (6) options.RNAseqThreshold = 0.2; (7) options.RNAsignThreshold = false; (8) options.VARfilter = false; (9)

options.VARscale = 'normal'; (10) options.VARperc = 50; (11) options.correctDistance = false; (12) options.calculateDS = true; (13) options.distanceCorrection = 'Euclidean'; (14) options.distanceThreshold = 2; (15) options.divideSamples = 'listCortex'; (16) options.excludeHippocampus = true; (17) options.Fit = {'exp'}; (18) options.normaliseWhat = 'Lcortex'; (19) options.normMethod = 'scaledRobustSigmoid'; (20) options.percentDS = 100; (21) options.doNormalise = true; (22) options.normaliseWithinSample = true; (23) options.meanSamples = 'meanSamples'; (24) options.parcellations = {'aparcaseg'}; (25) options.saveOutput = true.

References

Arnatkeviciute, A., Fulcher, B.D., and Fornito, A. (2019). A practical guide to linking brain-wide gene expression and neuroimaging data. *Neuroimage* 189**,** 353-367. doi: 10.1016/j.neuroimage.2019.01.011.

Fan, L., Li, H., Zhuo, J., Zhang, Y., Wang, J., Chen, L., et al. (2016). The Human Brainnetome Atlas: A New Brain Atlas Based on Connectional Architecture. *Cereb Cortex* 26(8)**,** 3508-3526. doi: 10.1093/cercor/bhw157.

Kang, H.J., Kawasawa, Y.I., Cheng, F., Zhu, Y., Xu, X., Li, M., et al. (2011). Spatio-temporal transcriptome of the human brain. *Nature* 478(7370)**,** 483-489. doi: 10.1038/nature10523.

2 Supplementary Figures and Tables

Table S1. Demographic data of subjects for each site
Table S2. MNI coordinates of the 29 core seeds in10 classic resting state networks
Table S3. Mean FD data in the total sample and subsamples
Fig. S1. Flowchart of subject inclusion and exclusion.
Fig. S2. Distributions of the regions of interest.
Fig. S3. Flowchart of tissue samples selection.
Fig. S4. The uncorrected case-control *t*-maps for the core seeds in auditory network.
Fig. S5. The uncorrected case-control *t*-maps for the core seeds in central executive network.
Fig. S6. The uncorrected case-control *t*-maps for the core seeds in dorsal attention network.
Fig. S7. The uncorrected case-control *t*-maps for the core seeds in default mode network.
Fig. S8. The uncorrected case-control *t*-maps for the core seeds in dorsal visual network.
Fig. S9. The uncorrected case-control *t*-maps for the core seed in primary visual network.
Fig. S10. The uncorrected case-control *t*-maps for the core seeds in sensorimotor network.
Fig. S11. The uncorrected case-control *t*-maps for the core seeds in salience network.
Fig. S12. The uncorrected case-control *t*-maps for the core seeds in ventral attention network.
Fig. S13. The uncorrected case-control *t*-maps for the core seed in ventral visual network.
Fig. S14. DFC differences between ASDs and TCs with 30 TRs and 70 TRs.
Fig. S15. Correlations between significant DFC changes in ASDs and symptom severity with 30 TRs.
Fig. S16. Correlations between significant DFC changes in ASDs and symptom severity with 70 TRs.
Fig. S17. Reoccurrence numbers of ΔDFC-related pathways (50 TRs) with 30 TRs and 70 TRs.
Fig. S18. DFC differences between ASDs and TCs in the total sample and subsamples with a median split based on FD.

2.1 Supplementary Tables

Table S1. Demographic data of participants for each site

GU	ASDs (n = 22) (Mean ± SD)	TCs (n = 21) (Mean ± SD)	p value
Age (years)	(8.64 - 13.88)<br>11.40 ± 1.41	(8.06 - 13.80)<br>10.90 ± 1.68	0.302
FIQ	117.68 ± 11.50	119.81 ± 10.67	0.534
Mean FD	0.132 ± 0.096	0.110 ± 0.094	0.444
ADOS-2 calibrated severity total score	5.80 ± 2.86<br>(n = 5)	-	-
SRS total score	84.00 ± 28.17<br>(n = 22)	19.67 ± 16.22<br>(n = 21)	< 0.001
SRS subscale score (raw)			
Awareness	10.95 ± 3.67	4.71 ± 3.13	< 0.001
Cognition	16.23 ± 5.02	2.57 ± 2.25	< 0.001
Communication	28.14 ± 11.12	6.57 ± 6.95	< 0.001
Motivation	13.23 ± 5.10	3.86 ± 3.41	< 0.001
Mannerisms	15.45 ± 6.52	1.95 ± 3.44	< 0.001
**NYU**	**ASDs (n = 20) (Mean ± SD)**	**TCs (n = 25) (Mean ± SD)**	**p value**
Age (years)	(5.43 - 17.93)<br>9.46 ± 3.19	(5.90 - 12.90)<br>9.19 ± 1.89	0.723
FIQ	112.85 ± 15.67	115.32 ± 13.14	0.568

Mean FD	0.105 ± 0.054	0.075 ± 0.042	0.045
ADOS-2 calibrated severity total score	6.10 ± 2.20 (*n* = 20)	-	-
SRS total score	82.85 ± 34.13 (*n* = 20)	22.38 ± 12.30 (*n* = 24)	< 0.001
SRS subscale score (raw)			
Awareness	10.80 ± 3.83	4.83 ± 2.46	< 0.001
Cognition	14.45 ± 6.81	3.17 ± 2.91	< 0.001
Communication	28.80 ± 13.56	6.96 ± 4.28	< 0.001
Motivation	13.65 ± 6.26	4.54± 3.08	< 0.001
Mannerisms	15.15 ± 7.51	2.88 ± 3.01	< 0.001
SDSU	ASDs (*n* = 17) (Mean ± SD)	TCs (*n* = 16) (Mean ± SD)	*p* value
Age (years)	(7.40 - 17.80) 12.90 ± 3.54	(8.10 - 17.60) 13.30 ± 3.09	0.732
FIQ	103.47 ± 8.70	104.56 ± 9.93	0.739
Mean FD	0.099 ± 0.074	0.071 ± 0.057	0.234
ADOS-2 calibrated severity total score	8.18 ± 1.91 (*n* = 17)	-	-
SRS total score	100.71 ± 26.48 (*n* = 17)	18.06 ± 10.84 (*n* = 16)	< 0.001
SRS subscale score (raw)			

Awareness	12.65 ± 4.21	3.88 ± 2.70	< 0.001
Cognition	15.94 ± 5.88	2.69 ± 2.47	< 0.001
Communication	33.53 ± 9.61	5.56 ± 4.55	< 0.001
Motivation	17.59 ± 5.04	3.75 ± 2.49	< 0.001
Mannerisms	21.00 ± 7.66	2.19 ± 2.81	< 0.001

SU	ASDs (n = 19) (Mean ± SD)	TCs (n = 15) (Mean ± SD)	p value
Age (years)	(8.43 - 12.99) 11.00 ± 1.12	(8.60 - 13.19) 11.24 ± 1.34	0.567
FIQ	112.37 ± 16.30	113.33 ± 11.79	0.8485
Mean FD	0.082 ± 0.034	0.082 ± 0.037	0.946
ADOS-2 calibrated severity total score	6.79 ± 1.62 (n = 19)	-	-
SRS total score	-	-	-
SRS subscale score (raw)			
Awareness	-	-	-
Cognition	-	-	-
Communication	-	-	-
Motivation	-	-	-

Mannerisms	-	-	-

TCD	ASDs (n = 10) (Mean ± SD)	TCs (n = 10) (Mean ± SD)	p value
Age (years)	(10.00 - 15.50) 12.65 ± 2.06	(12.00 - 17.50) 14.28 ± 1.72	0.071
FIQ	115.40 ± 11.42	120.60 ± 10.56	0.305
Mean FD	0.176 ± 0.096	0.096 ± 0.044	0.026
ADOS-2 calibrated severity total score	-	-	-
SRS total score	92.40 ± 24.56 (n = 10)	16.70 ± 15.03 (n = 10)	< 0.001
SRS subscale score (raw)			
Awareness	13.60 ± 3.78	2.80 ± 2.04	< 0.001
Cognition	18.00 ± 6.27	1.60 ± 2.46	< 0.001
Communication	32.10 ± 5.97	5.80 ± 5.39	< 0.001
Motivation	14.50 ± 6.31	4.40 ± 4.60	< 0.001
Mannerisms	14.20 ± 6.23	2.10 ± 2.51	< 0.001

Abbreviations: ADOS, Autism Diagnostic Observation Schedule; ASDs, autism spectrum disorders; FD, framewise displacement; FIQ, Full-scale Intelligence Quotient; GU, Georgetown University; NYU, NYU Langone Medical Center; SCQ, Social Communication Questionnaire; SD, standard deviation; SDSU, San Diego State University; SRS, Social Responsiveness Scale; SU, Stanford University; TCs, typical controls; TCD, Trinity College Dublin; p values were calculated by two-sample t-tests; -, not available.

Table S2. MNI coordinates of the 29 core seeds in 10 classic resting state networks

RSN	ROIs	MNI coordinates (x, y, z)
AN	STG	L (-44, -18, 5); R (45, -19, 6)
CEN	DLPFC	L (-45, 16, 45); R (45, 16, 45)
	PPC	L (-38, -53, 45); R (54, -50, 50)
DAN	FEF	L (-24, -7, 54); R (28, -7, 53)
	IPS	L (-27, -55, 56); R (31, -55, 55)
DMN	MPFC	(-1, 47, -4)
	PCC	(-5, -49, 40)
DVN	SOG	L (-37, -79, 10); R (38, -72, 13)
PVN	CF	(2, -79, 12)
SMN	PCG	L (-49, -2, 40); R (52, -4, 44)
	PoG	L (-46, -12, 38); R (42, -12, 34)
	SMA	L (-2, 4, 58); R (2, 6, 60)
SN	dACC	(4, 30, 30)
	FIC	L (-32, 24, -6); R (37, 25, -4)
VAN	OFC	L (-37, 27, -8); R (34, 27, -10)
	TPJ	L (-52, -53, 23); R (52, -52, 25)
VVN	CG	(0, -93, 4)

Abbreviations: AN, auditory network; CEN, central executive network; CF, calcarine fissure; CG, calcarine gyri; dACC, dorsal anterior cingulate cortex; DAN, dorsal attention network; DLPFC, dorsolateral prefrontal cortex; DMN, default mode network; DVN, dorsal visual network; FEF, frontal eye field; FIC, frontoinsular cortex; IPS, intraparietal sulcus; L, left; MPFC, medial prefrontal cortex; OFC, orbitofrontal cortex; PCC, posterior cingulate cortex; PCG, precentral gyrus; PoG, postcentral gyrus; PPC, posterior parietal cortex; PVN, primary visual network; R, right; ROIs, regions of interest; RSN, resting-state networks; SMA, supplementary motor area; SMN, sensorimotor network; SN, salience network; SOG, superior

occipital gyrus; STG, superior temporal gyrus; TPJ, temporoparietal junction; VAN, ventral attention network; VVN, ventral visual network.

Table S3. Mean FD data in the total sample and subsamples

Mean FD	ASDs (Mean ± SD)	TCs (Mean ± SD)	*p* value
ASDs vs. TCs	0.115 ± 0.073	0.090 ± 0.054	1.2e-2
ASDs with FD below the median vs. TCs with FD above the median	0.062 ± 0.016	0.123 ± 0.069	1.22e-7
ASDs with FD above the median vs. TCs with FD below the median	0.166 ± 0.078	0.050 ± 0.013	2.33e-15

Abbreviations: ASDs, autism spectrum disorders; FD, framewise displacement; SD, standard deviation; TCs, typical controls; *p* values were calculated by two-sample *t*-tests.

1.2 Supplementary Figures

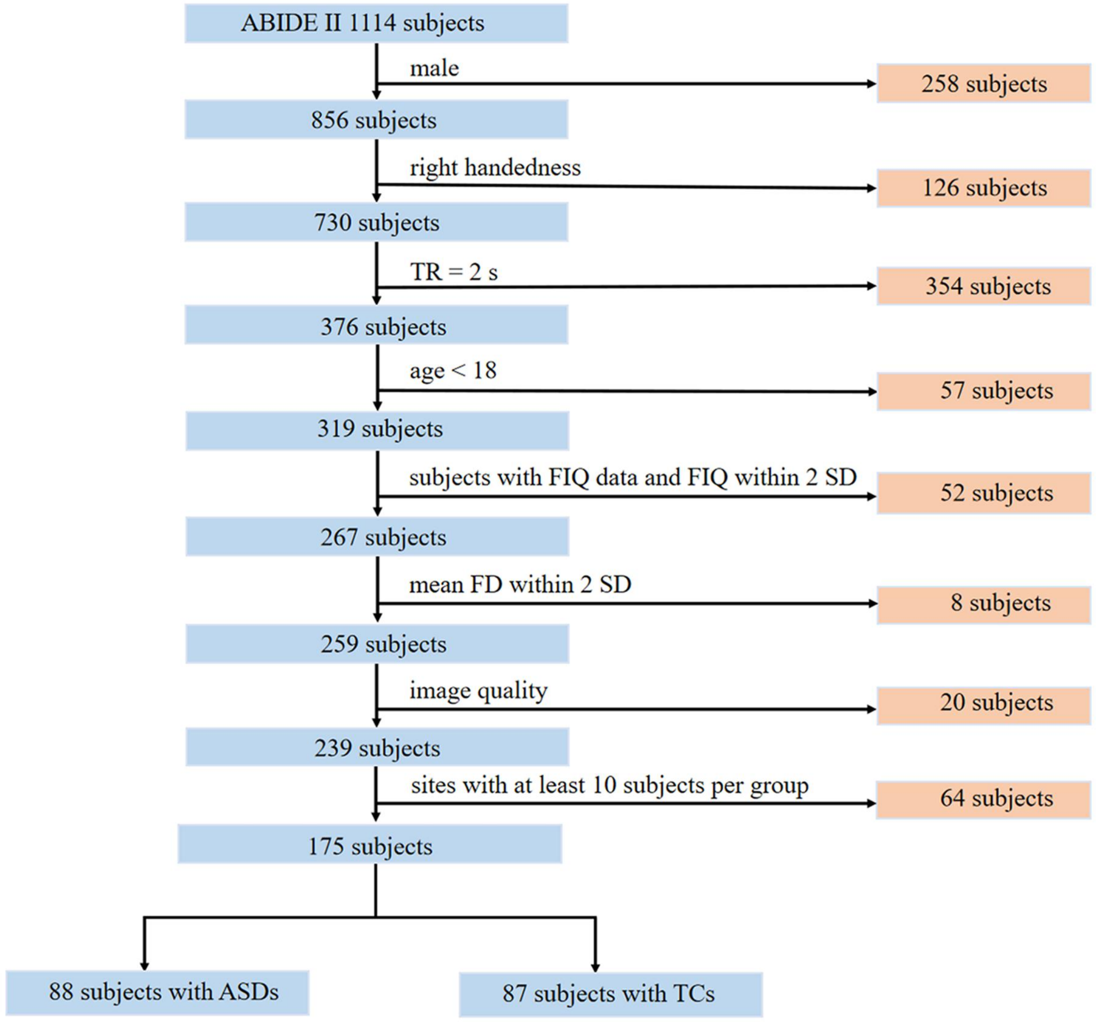

Fig. S1. Flowchart of subject inclusion and exclusion.
Abbreviations: ABIDE, Autism Brain Imaging Data Exchange; ASDs, Autism spectrum disorders; FD, framewise displacement; FIQ, full-scale Intelligence Quotient; SD, standard deviation; TCs, typical controls; TR, repetition time.

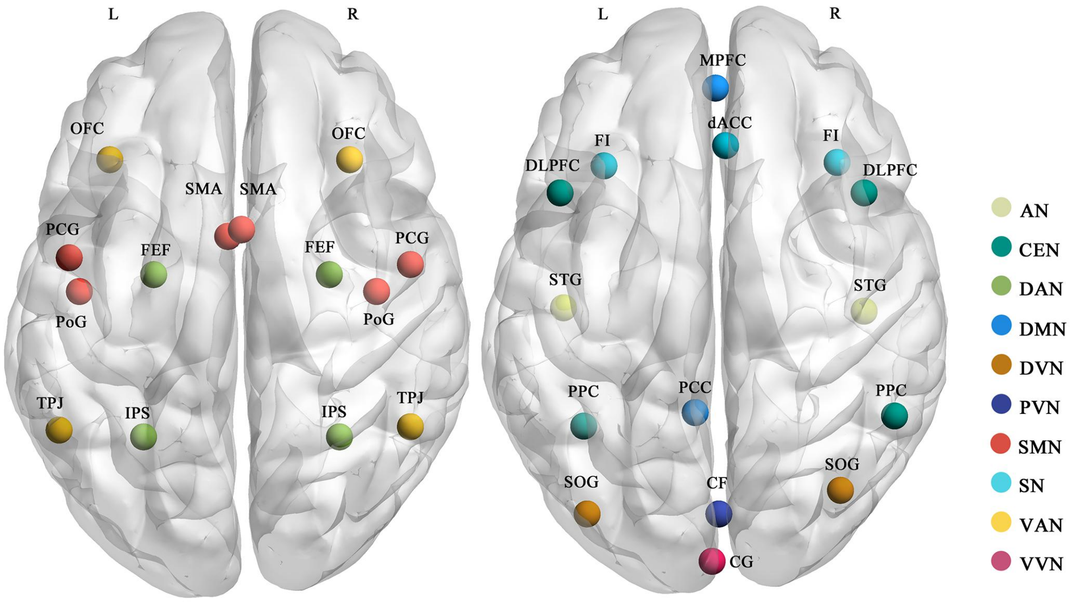

Fig. S2. Distributions of the ROIs. The core seeds within the same network are shown in the same color.

Abbreviations: AN, auditory network; CEN, central executive network; CF, calcarine fissure; CG, calcarine gyri; dACC, dorsal anterior cingulate cortex; DAN, dorsal attention network; DLPFC, dorsolateral prefrontal cortex; DMN, default mode network; DVN, dorsal visual network; FEF, frontal eye field; FIC, frontoinsular cortex; IPS, intraparietal sulcus; L, left; MPFC, medial prefrontal cortex; OFC, orbitofrontal cortex; PCC, posterior cingulate cortex; PCG, precentral gyrus; PoG, postcentral gyrus; PPC, posterior parietal cortex; PVN, primary visual network; R, right; SMA, supplementary motor area; SMN, sensorimotor network; SN, salience network; SOG, superior occipital gyrus; STG, superior temporal gyrus; TPJ, temporoparietal junction; VAN, ventral attention network; VVN, ventral visual network.

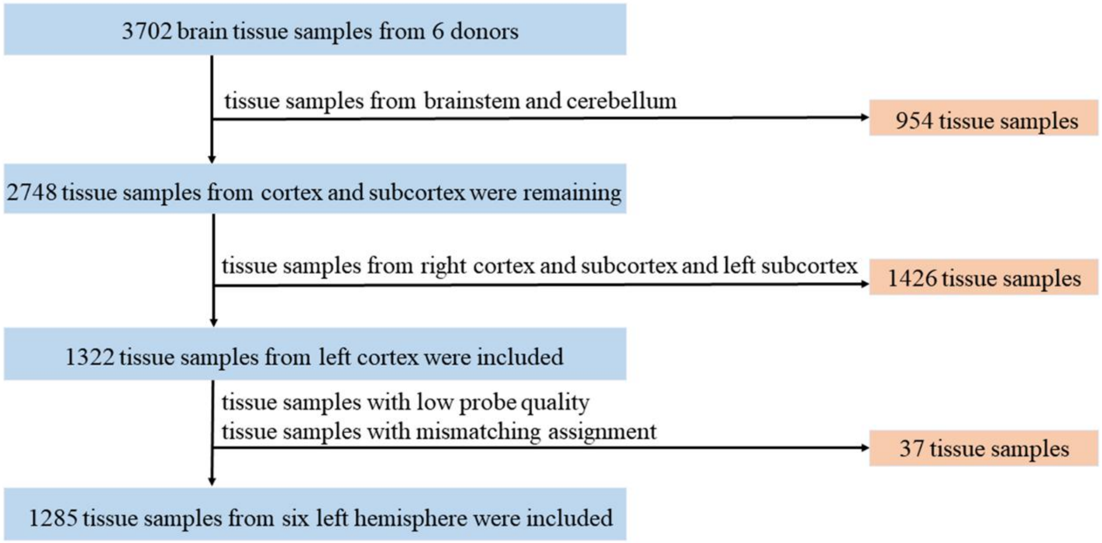

Fig. S3. Flowchart of tissue samples selection according to the reference (Arnatkeviciute et al., 2019).

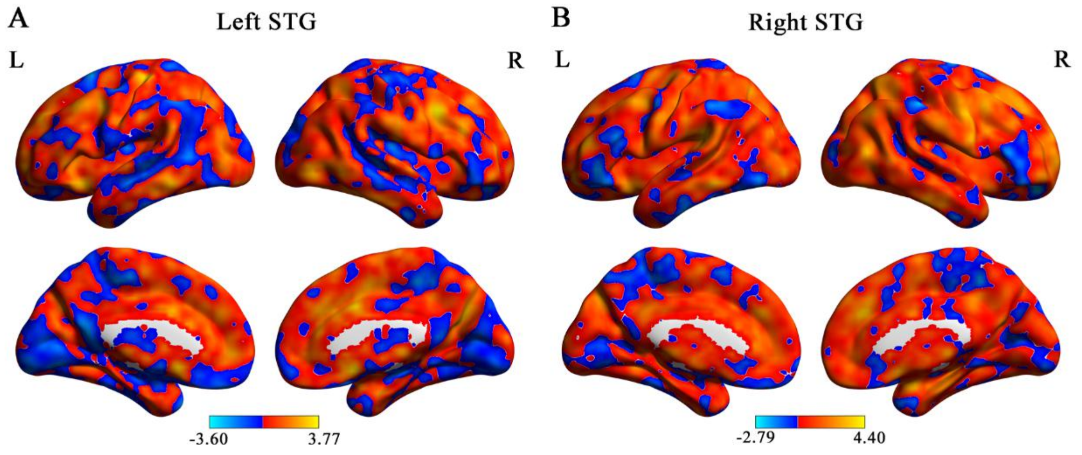

Fig. S4. The uncorrected case-control *t*-maps for the core seeds in auditory network.

The uncorrected case-control *t*-map of left STG (A) and right STG (B), respectively. The color bar represents *t*-statistic.

Abbreviations: L, left; R, right; STG, superior temporal gyrus.

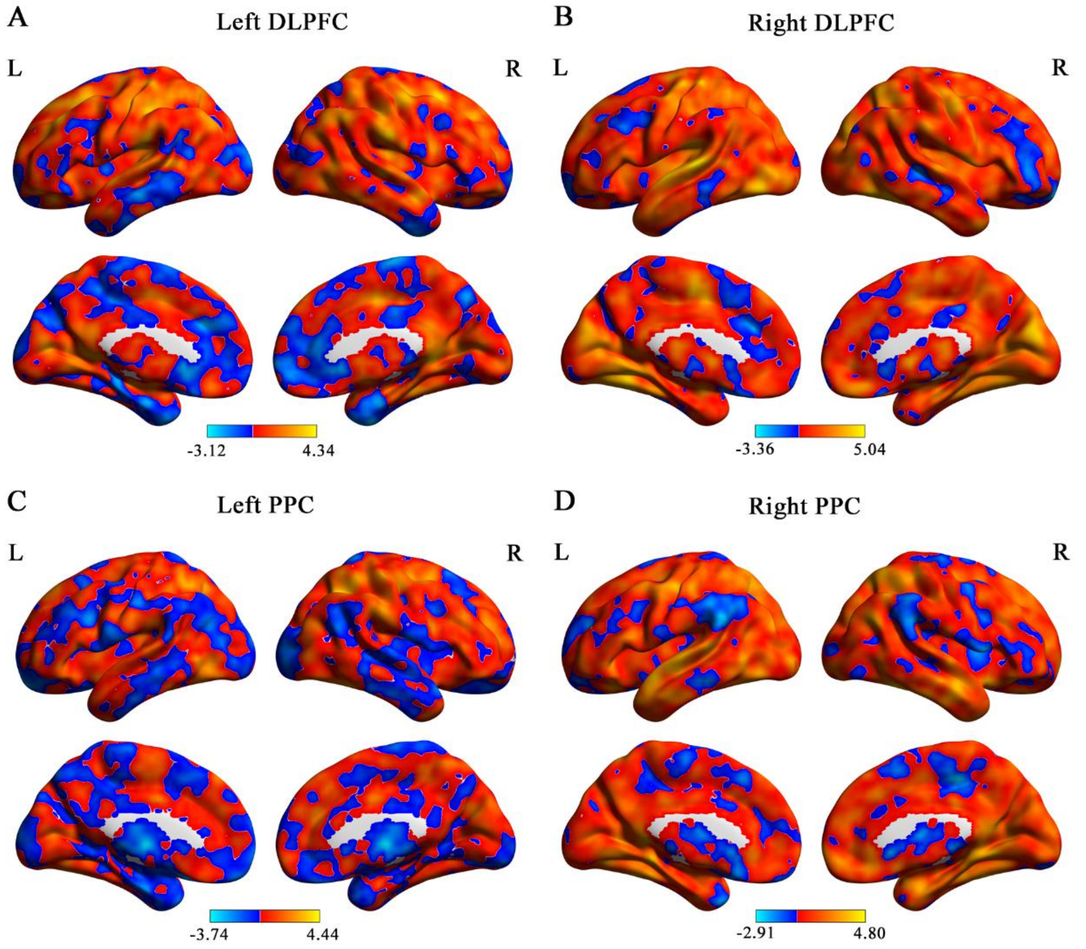

Fig. S5. The uncorrected case-control *t*-maps for the core seeds in central executive network.

The uncorrected case-control *t*-map of left DLPFC (A), right DLPFC (B), left PPC (C), right PPC (D), respectively. The color bar represents *t*-statistic.

Abbreviations: DLPFC, dorsolateral prefrontal cortex; L, left; PPC, posterior parietal cortex; R, right.

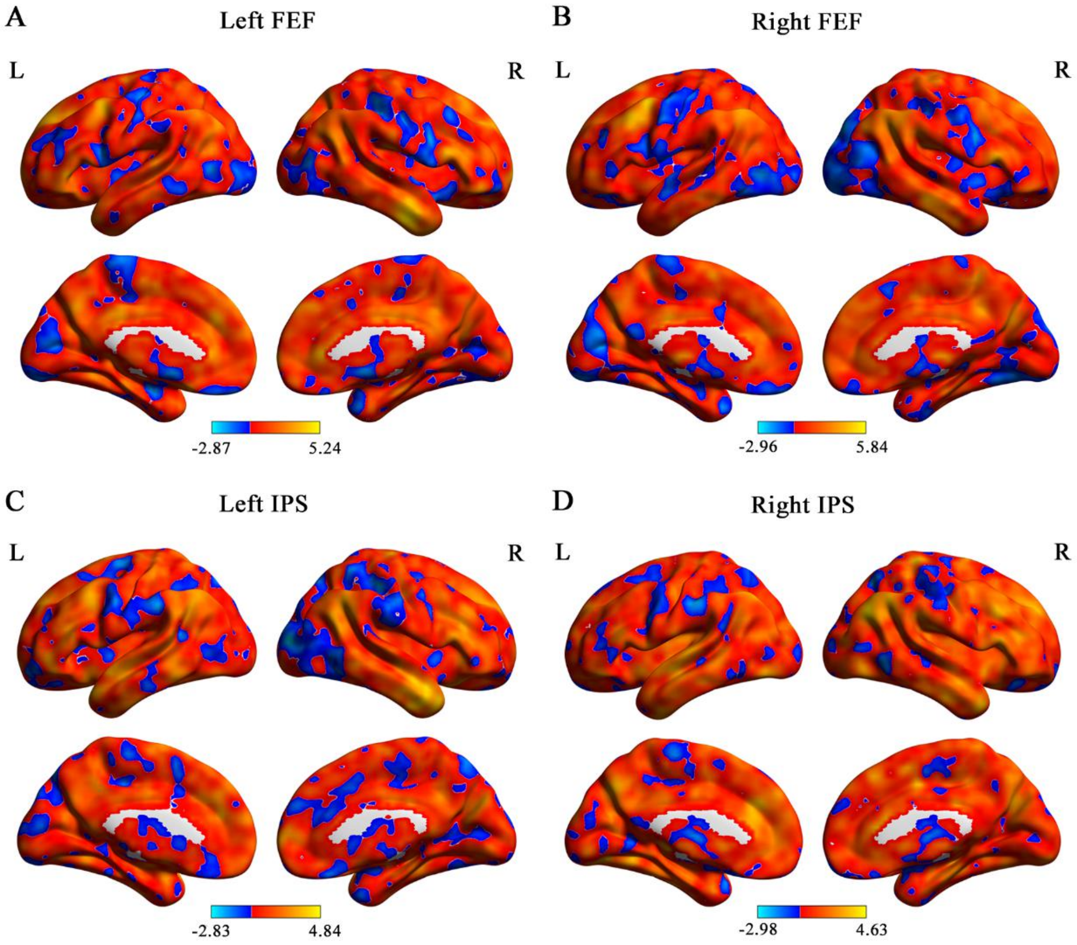

Fig. S6. The uncorrected case-control *t*-maps for the core seeds in dorsal attention network.

The uncorrected case-control *t*-map of left FEF (A), right FEF (B), left IPS (C), right IPS (D), respectively. The color bar represents *t*-statistic.

Abbreviations: FEF, frontal eye field; IPS, intraparietal sulcus; L, left; R, right.

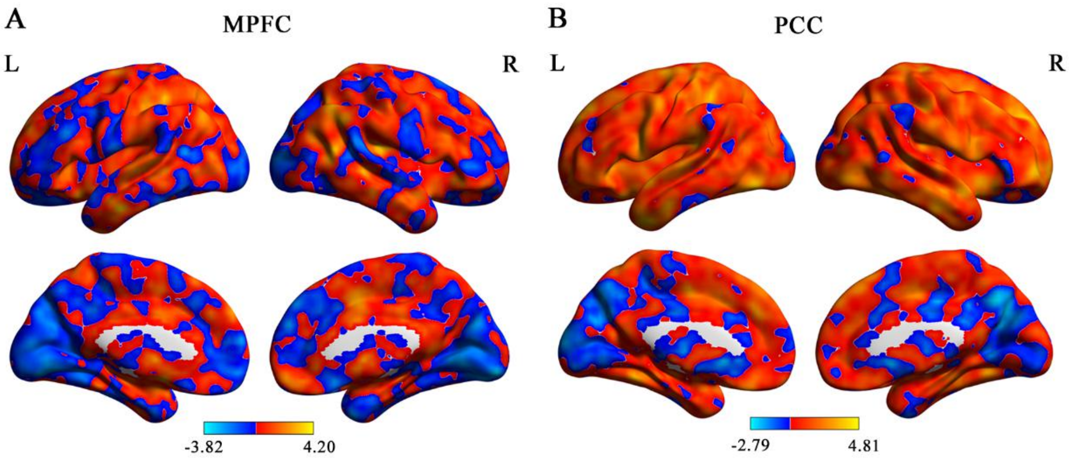

Fig. S7. The uncorrected case-control *t*-maps for the core seeds in default mode network.

The uncorrected case-control *t*-map of MPFC (A), MPFC (B), respectively. The color bar represents *t*-statistic.

Abbreviations: L, left; MPFC, medial prefrontal cortex; PCC, posterior cingulate cortex; R, right.

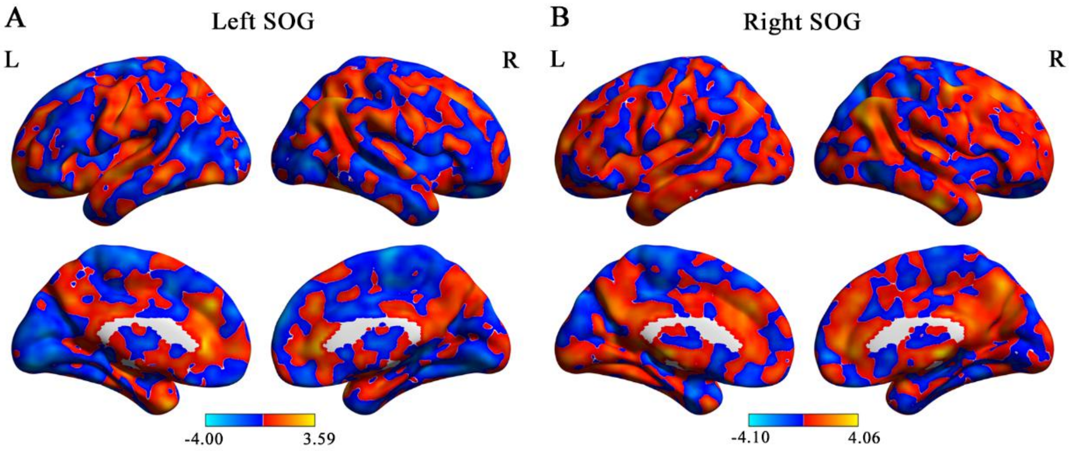

Fig. S8. The uncorrected case-control *t*-maps for the core seeds in dorsal visual network.

The uncorrected case-control *t*-map of left SOG (A), right SOG (B), respectively. The color bar represents *t*-statistic.

Abbreviations: L, left; R, right; SOG, superior occipital gyrus.

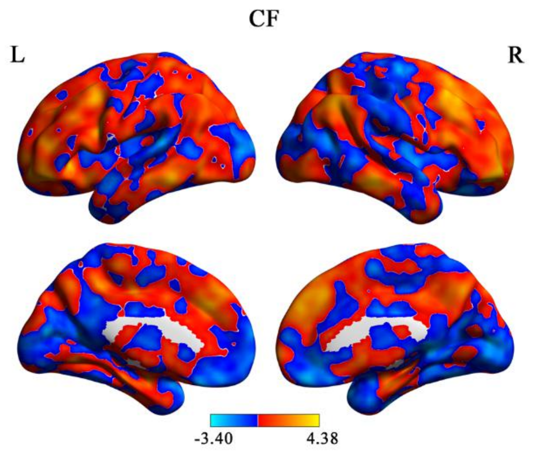

Fig. S9. The uncorrected case-control *t*-maps for the core seed in primary visual network.

The uncorrected case-control *t*-map of left CF. The color bar represents *t*-statistic.

Abbreviations: CF, calcarine fissure; L, left; R, right.

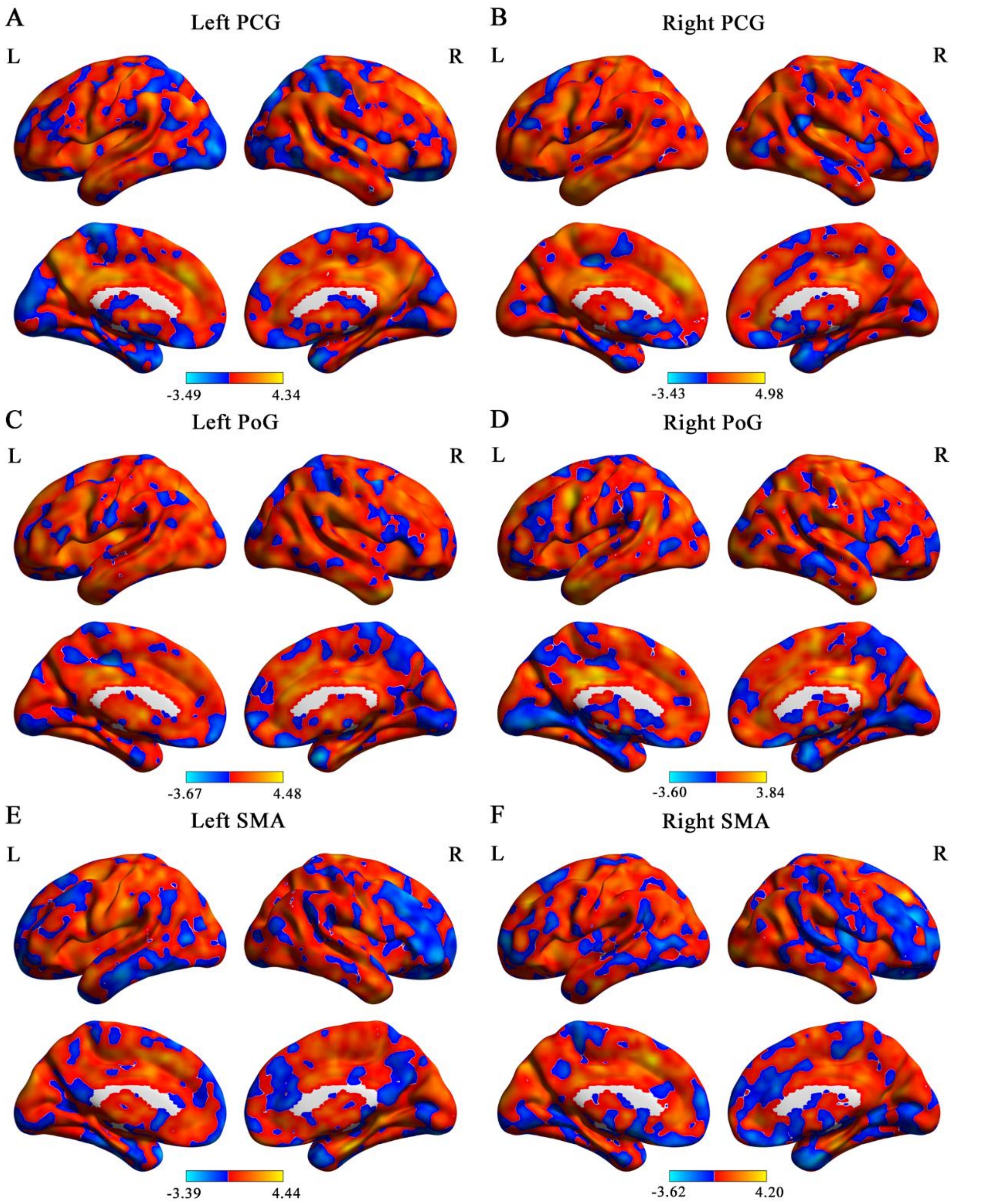

Fig. S10. The uncorrected case-control *t*-maps for the core seeds in sensorimotor network.

The uncorrected case-control *t*-map of left PCG (A), right PCG (B), left PoG (C), right PoG (D), left SMA (E), right SMA (F), respectively. The color bar represents *t*-statistic.

Abbreviations: L, left; PCG, precentral gyrus; PoG, postcentral gyrus; R, right; SMA, supplementary motor area.

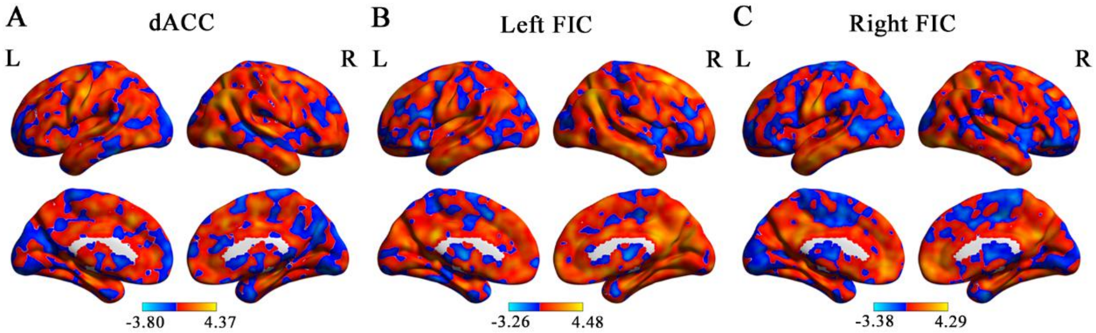

Fig. S11. The uncorrected case-control *t*-maps for the core seeds in salience network.

The uncorrected case-control *t*-map of dACC (A), left FIC (B), right FIC (C), respectively. The color bar represents *t*-statistic.

Abbreviations: dACC, dorsal anterior cingulate cortex; FIC, frontoinsular cortex; L, left; R, right.

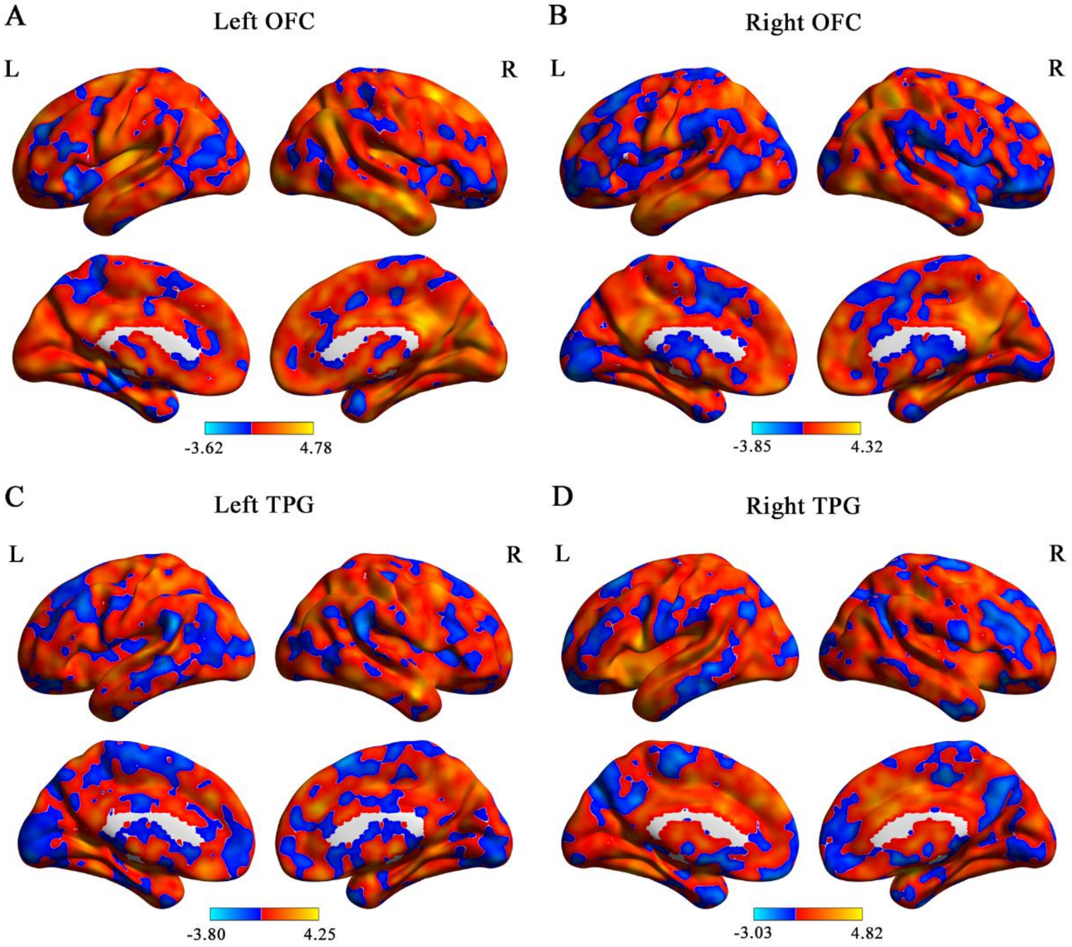

Fig. S12. The uncorrected case-control *t*-maps for the core seeds in ventral attention network.

The uncorrected case-control *t*-map of left OFC (A), right OFC (B), left TPG (C), right TPG (D), respectively. The color bar represents *t*-statistic.

Abbreviations: L, left; OFC, orbitofrontal cortex; R, right; TPJ, temporoparietal junction.

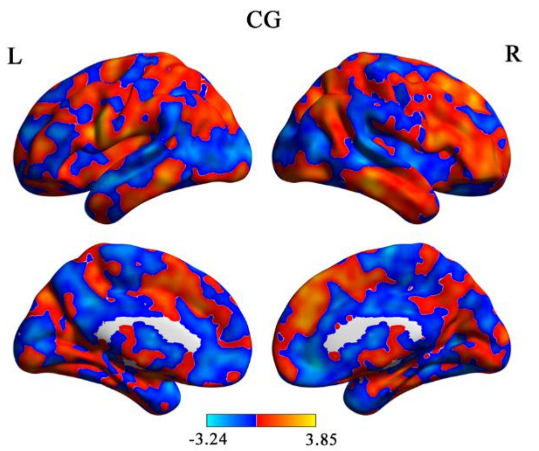

Fig. S13. The uncorrected case-control *t*-maps for the core seed in ventral visual network.

The uncorrected case-control *t*-map of CG. The color bar represents *t*-statistic.

Abbreviations: CG, calcarine gyri; L, left; R, right.

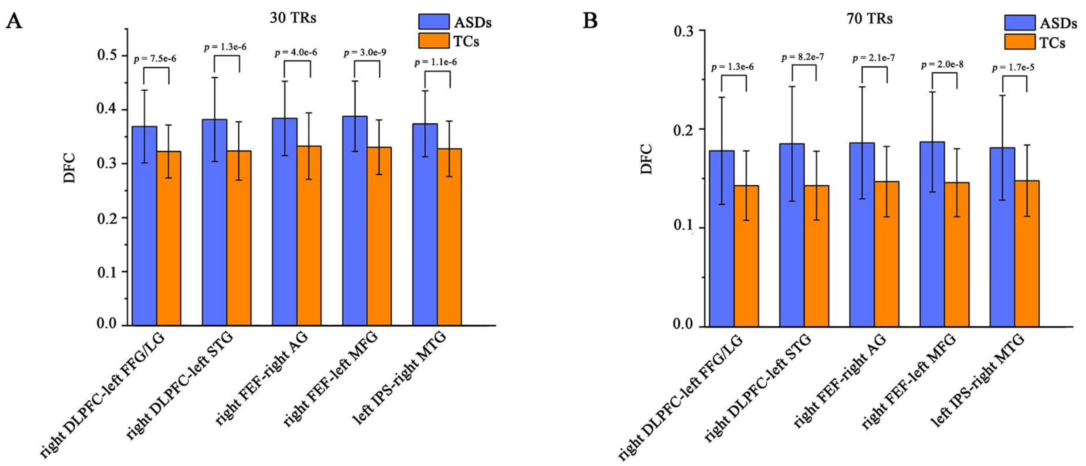

Fig. S14. DFC differences between ASDs and TCs with 30 TRs and 70 TRs. Bar graphs show the mean and standard deviation of the mean DFC value of each significant cluster in each group. The *p* values represent the significance of between-group difference obtained by general linear models while controlling for age, FIQ and mean FD.

Abbreviations: AG, angular gyrus; ASDs, autism spectrum disorders; DLPFC, dorsolateral prefrontal cortex; FEF, frontal eye field; FFG, fusiform gyrus; IPS, intraparietal sulcus; LG, lingual gyrus; MFG, middle frontal gyrus; MTG, middle temporal gyrus; STG, superior temporal gyrus; TCs, typical controls.

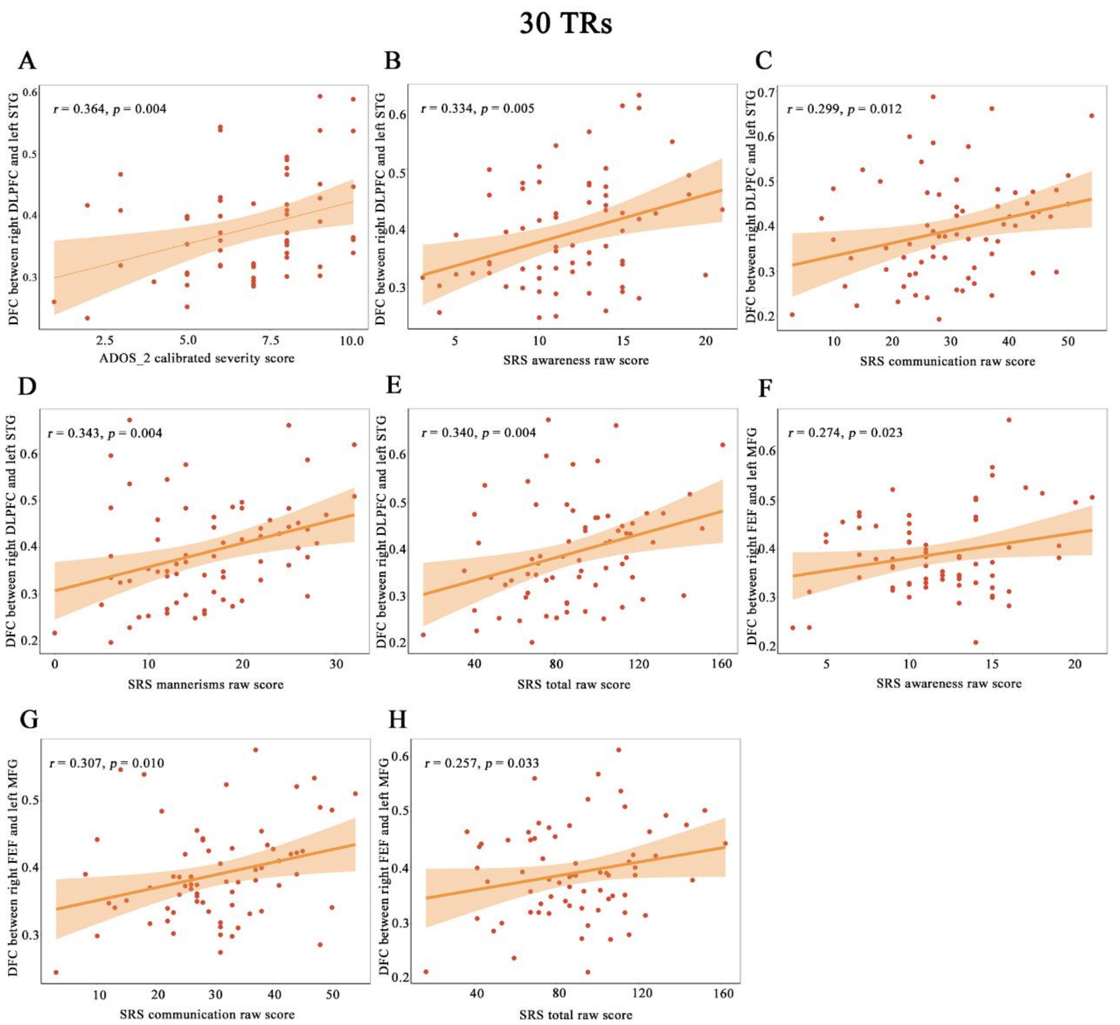

Fig. S15. Correlations between significant DFC changes in patients with ASDs and symptom severity with 30 TRs.

The correlation between the DFC of the right DLPFC with the left STG and with ADOS_2 calibrated severity score (A), SRS awareness raw score (B), SRS communication raw score (C), SRS mannerisms raw score (D) and SRS total raw score (E). The correlation between the DFC of the right FEF with the left MFG and with SRS awareness raw score (F), SRS communication raw score (G) and SRS total raw score (H). Shades represent the 95% confidence intervals.

Abbreviations: ADOS, Autism Diagnostic Observation Schedule; DLPFC, dorsolateral prefrontal cortex; FEF, frontal eye field; FFG, fusiform gyrus; LG, lingual gyrus; MFG, middle frontal gyrus; SRS, Social Responsiveness Scale; STG, superior temporal gyrus; *p*, uncorrected *p* value.

70 TRs

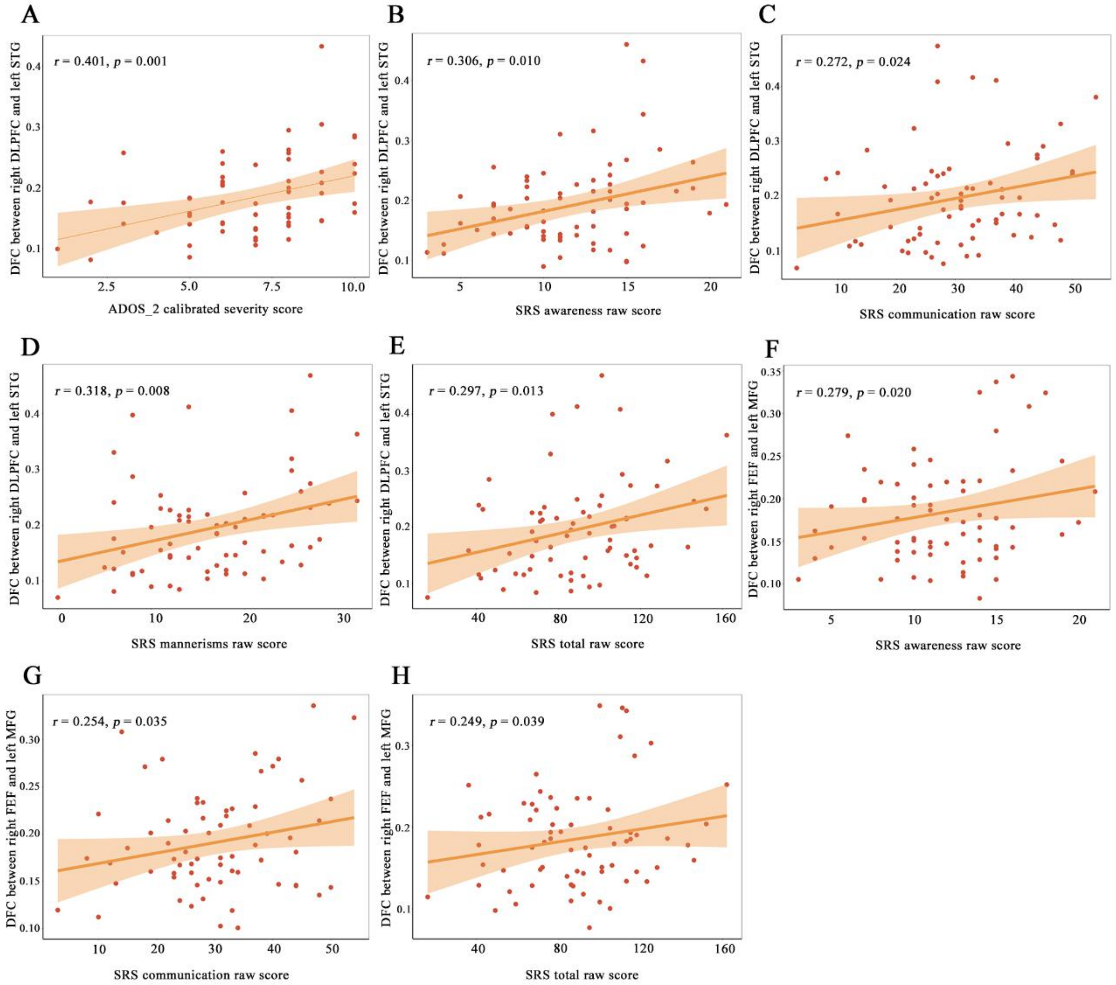

Fig. S16. Correlations between significant DFC changes in patients with ASDs and symptom severity with 70 TRs.

The correlation between the DFC of the right DLPFC with the left STG and with ADOS_2 calibrated severity score (A), SRS awareness raw score (B), SRS communication raw score (C), SRS mannerisms raw score (D) and SRS total raw score (E). The correlation between the DFC of the right FEF with the left MFG and with SRS awareness raw score (F), SRS communication raw score (G) and SRS total raw score (H). Shades represent the 95% confidence intervals.

Abbreviations: ADOS, Autism Diagnostic Observation Schedule; DLPFC, dorsolateral prefrontal cortex; FEF, frontal eye field; FFG, fusiform gyrus; LG, lingual gyrus; MFG, middle frontal gyrus; SRS, Social Responsiveness Scale; STG, superior temporal gyrus; *p*, uncorrected *p* value.

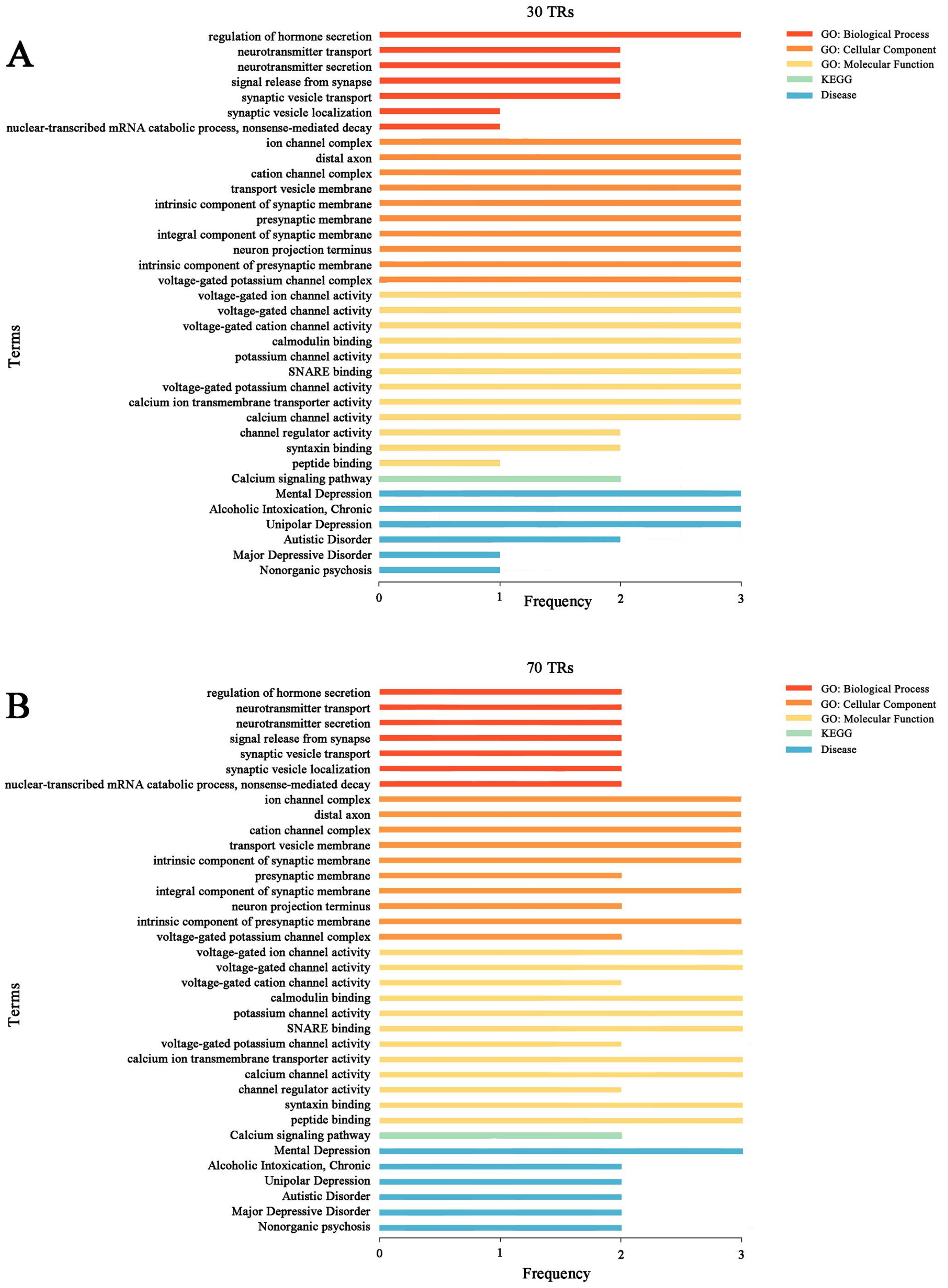

Fig. S17. Reoccurrence numbers of ∆DFC-related pathways (50 TRs) with 30 TRs and 70 TRs.

Three transcription-neuroimaging association analyses were conducted because three *t*-maps exhibited significant between-group differences (see the **Results** section in the Main Text), and the frequency of occurrence of the significant terms in the main results was calculated.

Abbreviations: GO: Gene Ontology; KEGG, Kyoto Encyclopedia of Genes and Genomes.

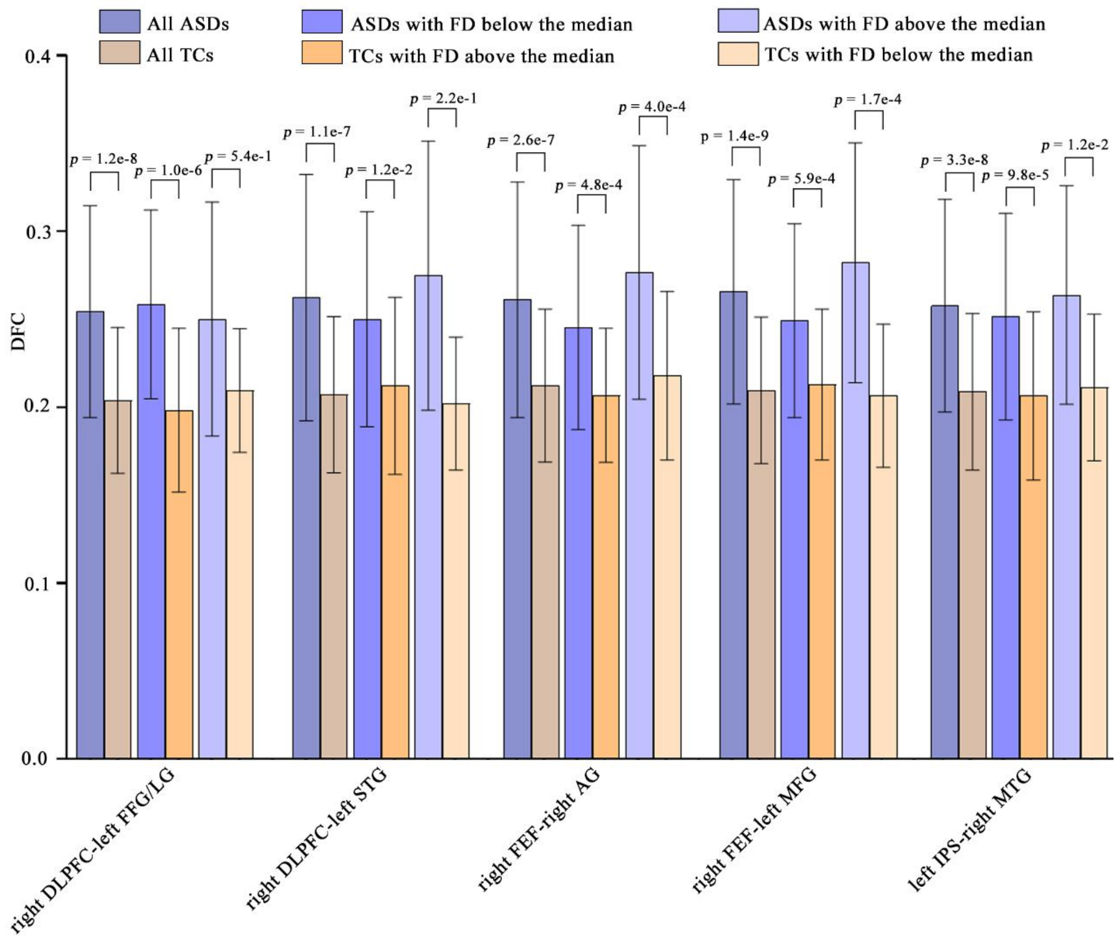

Fig. S18. DFC differences between ASDs and TCs in the total sample and subsamples with a median split based on FD. Bar graphs show the mean and standard deviation of the mean DFC value of each significant cluster in each group. The *p* values represent the significance of between-group difference obtained by general linear models while controlling for age, FIQ and mean FD.

Abbreviations: AG, angular gyrus; ASDs, autism spectrum disorders; DLPFC, dorsolateral prefrontal cortex; FD, framewise displacement; FEF, frontal eye field; FFG, fusiform gyrus; IPS, intraparietal sulcus; LG, lingual gyrus; MFG, middle frontal gyrus; MTG, middle temporal gyrus; STG, superior temporal gyrus; TCs, typical controls.
